# Computer mouse tracking reveals motor signatures in a cognitive task of spatial language grounding

**DOI:** 10.3758/s13414-019-01847-9

**Published:** 2019-09-12

**Authors:** Jonas Lins, Gregor Schöner

**Affiliations:** Institut für Neuroinformatik, Ruhr-Universität Bochum, Universitätsstraße 150, 44801 Bochum, Germany

**Keywords:** Embodied perception, Goal-directed movements, Perception and action

## Abstract

In a novel computer mouse tracking paradigm, participants read a spatial phrase such as “The blue item to the left of the red one” and then see a scene composed of 12 visual items. The task is to move the mouse cursor to the target item (here, blue), which requires perceptually grounding the spatial phrase. This entails visually identifying the reference item (here, red) and other relevant items through attentional selection. Response trajectories are attracted toward distractors that share the target color but match the spatial relation less well. Trajectories are also attracted toward items that share the reference color. A competing pair of items that match the specified colors but are in the inverse spatial relation increases attraction over-additively compared to individual items. Trajectories are also influenced by the spatial term itself. While the distractor effect resembles deviation toward potential targets in previous studies, the reference effect suggests that the relevance of the reference item for the relational task, not its role as a potential target, was critical. This account is supported by the strengthened effect of a competing pair. We conclude, therefore, that the attraction effects in the mouse trajectories reflect the neural processes that operate on sensorimotor representations to solve the relational task. The paradigm thus provides an experimental window through motor behavior into higher cognitive function and the evolution of activation in modal substrates, a longstanding topic in the area of embodied cognition.

## Introduction

Most everyday tasks are a seamless combination of perception, cognition, and action. To pick a snack at a self-service bakery, I have to recognize the different varieties of pastry on the counter, decide which one I like best, and reach for it. Classical theories of the human mind hold that these different processes occur in sequence: perceiving, deciding, acting (Newell, 1990). Intuitively, however, it feels that these things may overlap in time. When I am rushed, I might start reaching before I know which pastry exactly I will pick, deciding as I go, and in effect my hand may follow a less-than-straight path as donuts are weighed against nearby croissants (Truong et al., 2013).

In line with this intuition, psychological researchers increasingly agree that the neural processes underlying perception, cognition, and action are closely interlinked (e.g., Pezzulo & Cisek 2016) and evolve in a graded and temporally continuous manner, rather than being strictly separable into sequential stages. An important source of support for this view comes from behavioral experiments in which motoric responses are influenced in a graded way by properties of perceptual or cognitive components of the task (Spivey, 2007).

### Trajectory attraction to non-target objects in visual space

Motor plans evolve continuously over time. Ghez et al., (1997) provided evidence for this view in their timed-movement-initiation paradigm, in which the time between a cue and movement initiation is systematically varied. For short stimulus-response intervals, movements fell close to a “default” direction that reflected the average target location. Response distributions gradually migrated toward the cued target with increasing stimulus-response interval. Subsequent research has substantiated this evidence both at the behavioral and the neural level. For instance, when the final target in an array of potential reaching goals is cued only upon movement onset, pointing trajectories curve toward the other items. The strength of attraction depends on the items’ spatial distribution, with multiple non-targets on one side of the display exerting stronger attraction than single ones (Gallivan and Chapman, 2014; Chapman et al., 2010). The visual saliency of potential targets strongly modulates trajectory bias, with highly salient potential targets attracting trajectories even when the opposite direction of curvature is predicted from the spatial distribution of potential targets (Wood et al., 2011). This suggests that there is a link between attentional deployment and motor planning. Song & Nakayama (2006; see also, 2007) used movement trajectories to capture attentional deployment when an odd-colored target had to be found among uniformly colored distractors. Attraction toward distractors was strong for one target with two distractors, but disappeared when attentional deployment to the target was made easier by increasing the number of uniformly colored distractors, enabling perceptual grouping, or by keeping target color fixed across trials. Similarly, attention-capturing motion at the location of a distractor increases attraction (Moher et al., 2015).

The dynamic neural field model of Erlhagen and Schöner (2002) postulates that values of motor parameters such as movement direction are represented as hills of localized activation within populations of neurons that are tuned to the parameters. Task demands or potential targets preactivate neurons in the distribution and interact with the input from the cued target. The model predicts the time course of activation in the population distribution from the early preshape (or default distribution) to the late form in which activation is centered on the cued target, accounting for the behavioral patterns observed by Ghez et al., (1997). The model predicts that the metrics of potential targets matter. Large differences between movement directions for the different targets lead to bimodal distributions of reaching directions at short stimulus-response intervals, which become monomodal over time. Small differences between the movement directions for different targets lead to monomodal distributions at all stimulus-response intervals, that merely shift toward the cued target. This dependence of response distributions on the metrics of the target set was also observed by Ghez et al., (1997).

The graded and time-continuous evolution of motor plans can be directly observed at the neural level by recording from populations of neurons in motor and premotor cortex. Monkeys reaching from a central button to one of six peripherally arranged target buttons were given varying amounts of prior information about the upcoming movement (Bastian et al. 1998, 2003). Distributions of population activation that represented the planned movement direction were observed during the delay between this prior information and the cue. Over time, the population shifted from an early, broad peak centered on the range of precued movement directions to a narrower peak centered on the movement direction to the specified target. Cisek and Kalaska (2005) performed the same experiment in which two precued targets implied movement directions that were 180 degrees apart. Now the early distribution of population activation was bimodal, switching to monomodal after the cue.

### Trajectory attraction based on abstract cognitive tasks

The studies reviewed above involve specification of movement targets directly through visual cues of varied timing, salience, and validity. Their influence on movement is accounted for by inputs to neural representations over a space of movement parameters that map one-to-one onto movement targets.

A related type of study involving computer mouse tracking (Spivey et al., 2005; Freeman et al., 2011) uses biases in response trajectories to gain insight into the evolution of decisions in more abstract spaces whose neural representations do not necessarily map directly onto the sensorimotor surfaces. In a typical mouse tracking experiment (e.g., Coco & Duran, 2005; Dale, Kehoe, & Spivey, 2007; Dale, Kehoe, & Spivey, 2008; Freeman, Ambady, Rule, & Johnson, 2009; Spivey et al., 2016), participants solve some abstract cognitive task, such as categorizing an animal name as referring to a mammal or non-mammal (Dale et al., 2007). They respond by moving a mouse-controlled cursor from a start location on the computer screen (typically a center-bottom location) to an appropriate response button (typically two buttons in the upper left and upper right of the screen). The trajectory of the mouse cursor is recorded and analyzed metrically.

The possible responses to the cognitive task are mapped onto the response buttons in an arbitrary manner (e.g., through verbal instruction or written labels). Deviations of the mouse trajectories from a straight path to the correct response button in the direction of the alternative response button are used to infer how the certainty about the cognitive decision evolves in time. More difficult decisions are commonly associated with stronger curvature than easier ones, so that the cursor bends toward the correct button later in its path. The trajectories are taken to reveal the moment-to-moment decision state of the cognitive system, reflecting the ongoing competition between response alternatives (Freeman et al., 2011).

This paradigm has been used for a range of high-level cognitive tasks, such as social categorization (Freeman et al., 2008, 2013; Freeman & Ambady 2011; Cloutier et al., 2014), processing of grammatical aspect (Anderson et al., 2013), vowel discrimination (Farmer et al., 2009), cognitive flexibility (Dshemuchadse et al., 2015), intertemporal decision-making and delay discounting (Dshemuchadse et al., 2013; Scherbaum et al., 2013, 2016), multitasking (Scherbaum et al., 2015), stimulus-response compatibility (Flumini et al., 2014), lexical decision (Barca & Pezzulo, 2012), and response selection (Wifall et al., 2017). The vast majority of mouse-tracking studies employed the standard two-choice paradigm (Hehman et al., 2015), although some variants have been explored, mostly in a similar methodological frame (e.g., Anderson et al., 2013; Cloutier et al., 2014; Farmer, Anderson, & Spivey, 2007; Farmer, Anderson, & Spivey, 2007; Scherbaum et al., 2013, 2017; Koop & Johnson, 2011).

### The current study

There are thus two broad categories of factors beyond the spatial attributes of an ultimate movement target that have been shown to influence the shape of motor responses. One category includes processes at the sensorimotor surfaces, evoked, for instance, by competing targets or salient distractors. The other one includes abstract cognitive tasks that evolve within neural domains remote from the sensorimotor level and that modulate motor decisions through learned links between actions and candidate solutions.

We aim to complement these previously studied factors with one that lies at the interface of high-level cognition and immediate perception. The experiments described here show that motor action is also influenced by attentional processes on a perceptual level that are integral components of a more abstract and complex cognitive task. We thus aim to observe signatures of the cognitive task directly, in an embodied and ecologically valid experimental setup where the cognitive task serves its proper role: using spatial language to identify objects in the world and thereby select movement targets.

Although we hope to reach situated cognition in general, our entry point is thus the “perceptual grounding” of spatial language in visual scenes. This task is sufficiently simple to be open to direct experimental assessment through movement, while also tapping into relational thinking, which implies a certain level of cognitive abstraction. Specifically, we ask participants to perceptually ground phrases about spatial relations such as “The green item to the left of the red one” by moving a cursor to the target that matches this description.

We have recently presented a neural process model that implements a neural mechanism for perceptual grounding of spatial and movement relations (Richter et al., 2014a, b, 2017). The model captures the processing steps that unfold in time when a relational phrase is linked to a visual scene. The structure of that model provided the heuristics for both the design and the expected outcome of the experiments we report. Before describing the experiments, we will therefore briefly summarize previous research into spatial language processing and sketch the neural model of relational grounding.

#### Spatial language

**Fig. 1:**
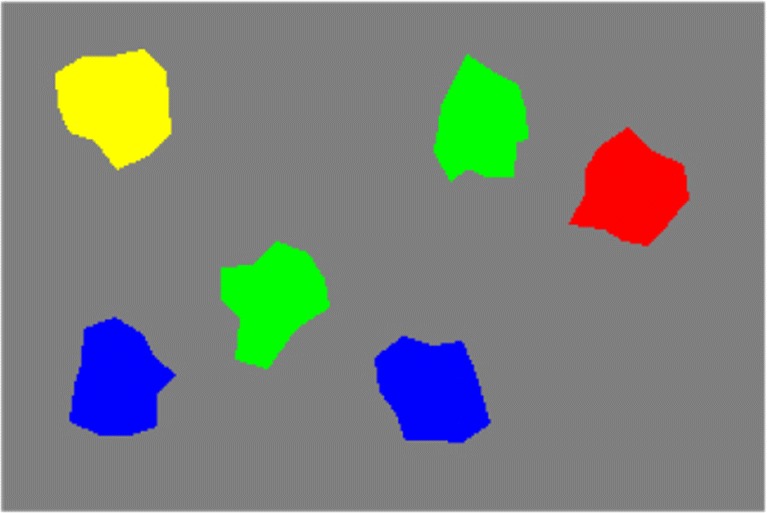
Referring to a particular blue or green item in this visual scene requires the use of spatial language. The scene was used as visual input to the model of spatial language grounding

Spatial language helps disambiguate referent objects when feature-based language is insufficient. “The blue object”, for instance, may refer to either of two objects in Fig. 1 while “the blue object to the right of the green object” uniquely specifies a single object. Relational phrases like this consist of three components: a *target object*, corresponding to the blue object in the example; the relation itself, denoted by the *spatial term* (“right” in the example); and a *reference object*, which corresponds to the green object in the example. We focus on the deictic relations left, right, above, and below.

Linking a spatial phrase that describes a deictic relation to a configuration of objects in the visual environment requires multiple computational steps, as analyzed by Logan and Sadler (1996). First, the two arguments of a relation must be linked to the locations of the corresponding objects in a perceptual representation. Logan and Sadler (1996) call this spatial indexing. Second, the parameters of the reference frame must be set. For deictic relations, the origin of the reference frame is centered on the reference object, while its other parameters, including scale, direction, and orientation, remain congruent with the viewer’s reference frame. Third, a spatial template must be imposed on the reference object within the adjusted reference frame. This template is specific to the relation in question and indicates the goodness of fit for different locations in space relative to the reference object. Finally, the goodness of fit must be assessed for the target object by matching its position to the spatial template.

It is evident from this framework that spatial relations are not instantly available throughout the visual field, which is likewise suggested by the combinatorial explosion of possible relations when many objects are present (Franconeri et al., 2012). In line with this, empirical evidence suggests that evaluating visual relations involves the sequential processing of objects and relational pairs. Most importantly, the classical notion that localizing features in the visual environment requires focused attention (Treisman & Gelade, 1980) entails that spatial indexing does so as well. This is backed up by a stronger engagement of selective attention when locations of visual targets are to be reported rather than merely detected (Hyun et al., 2009). EEG data likewise support this conclusion. When participants saw two visual stimuli and judged their spatial relation, EEG showed attention shifts between them, despite the instruction to focus on both items at the same time, showing that either stimulus needed to be sequentially selected to evaluate their relation (Franconeri et al., 2012).

Eye-tracking data further highlight the role of attentional selection in establishing reference between linguistic input and visual scenes, particularly for spatial language (Eberhard et al., 1995; Tanenhaus et al., 1995). Yuan et al., (2016) briefly presented participants with visual displays of two stimuli that were vertically aligned and could thus be viewed as instantiating an ‘above’ or ‘below’ relation. Participants had to indicate for a queried item whether it had been in the upper or lower position. If a saccade from the non-queried to the queried item had occurred, responses were faster than when the other item was queried, suggesting that sequential order may play a role in judging relations. Another eye-tracking study using relations between object pictures showed similar gaze shifts (Burigo and Knoeferle, 2015), albeit without fully settling the role of shift order (and modeling efforts are similarly inconclusive in this respect; Kluth, Burigo, & Knoeferle, 2001; Regier & Carlson, 2016).

Sequential processing is furthermore induced by the presence of multiple candidate pairs. In visual search experiments by Logan (1994; see also, Moore, Elsinger, & Lleras, 2001; for review, see Carlson & Logan, 2005) participants saw visual displays with multiple item pairs and reported the presence or absence of a target pair that was defined by a relational phrase (e.g., by “dash above plus”) and placed among distractor pairs which instantiated the opposite relation (e.g., dashes below pluses). Search time rose steeply with the number of distractor pairs. Search time slopes were flat, in contrast, when distractor pairs consisted of all dashes or all pluses, attributed to pop-out of the discrepant item in the target pair (Logan, 1994). The pop-out did not appear to help processing the relation of the pair, however, deciding whether the sought relation was present still took more time than only deciding whether a discrepant item was present (probed in another condition). Thus, attentional allocation is required but not sufficient to process relations, which instead seems to involve additional steps (Logan, 1994).

Together, the evidence suggests that sequential selection of visual items plays an important role in multiple stages of relational processing, although leaving some open questions with respect to the underlying mechanisms.

#### A dynamic neural field model of spatial language grounding

We provide a rough outline of the model, which is presented in detail elsewhere (Richter, Lins, Schneegans, Sandamirskaya, & Schöner, 2014a; see also, 2017, Richter et al., 2014b). The model is framed in dynamic field theory (DFT; Lins & Schöner, 2014; Schöner, 2015; Schöner, Spencer, & the DFT Research Group, 2008), a set of concepts that neurally ground perceptual, motor, and cognitive processes. In DFT, distributions of activation over populations of neurons are modeled and simulated as dynamic neural fields, which are defined over the continuous metric dimensions that the modeled populations are sensitive to, such as retinal space, color, or movement space. This reflects the tuning of neural activity to input or output dimensions (see Bastian et al., 2003; Erlhagen, Bastian, Jancke, Riehle, & Schöner, 1999; Jancke et al., 1999, for the neurophysiological foundation of DFT).

Dynamic neural fields evolve continuously in time. They receive input from the sensory surfaces or through synaptic connections from other dynamic fields. An object in the visual array, for instance, may induce a localized bump of activation in a field defined over retinal space. If such input is strong enough to push activation across a threshold, output is generated, and a localized peak of activation may arise. The output may impact other fields or motor systems via synaptic connections. On the other hand, output also drives lateral interaction between different sites within the same field: Neighboring sites excite each other (local excitation) while remote sites inhibit each other (surround inhibition). When this recurrent regime is entered, the emerging peak is to a degree decoupled from the input and thus stabilized against input fluctuations or other perturbations (which ultimately enables stable cognition in situated agents; Lins & Schóner, 2014; Schöner, 2008; Schöner et al. 2015). Peak formation in DFT thus represents an elementary decision about the presence or computational relevance of what brought it about.

**Fig. 2:**
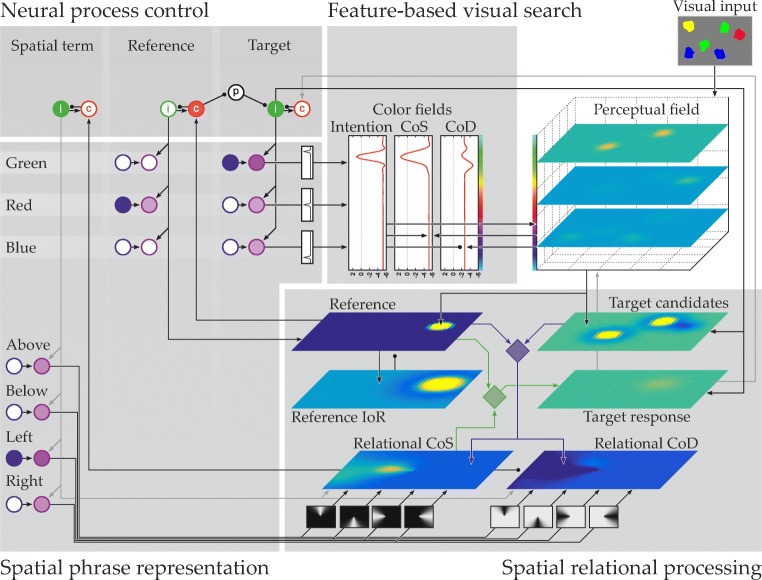
The dynamic field model of spatial language grounding. Main components referred to in the text are indicated by gray boxes

The neural process model of the perceptual grounding of spatial language is a seamless dynamical system composed of multiple interconnected fields that implement mechanisms of scene representation, visual search, spatial phrase representation, neural process control, and relational processing (Fig. 2). The spatial phrase representation (left in Fig. 2) guides processes in the sensory parts of the architecture that receive input from a visual image. The visual image is supplied to the *perceptual field*, shown at the top right of Fig. 2. This field is defined over two dimensions of image space and one color dimension. Visual items initially lead to hills of sub-threshold activation in the perceptual field. The locations of these hills along the field’s dimensions indicate colors and spatial positions of the items. The perceptual field is the visual scene representation to which other parts connect in order to drive attention for visual search or to receive location or color input for further processing.

To ground a spatial phrase such as “The green item to the left of the red item” in a scene like Fig. 1, the phrase is stored in the spatial phrase representation. In a first step, then, the component for feature-based visual search drives feature attention in the perceptual field, bringing all items in the reference color (here, red) above the output threshold. The output is projected to the relational component (bottom right in Fig. 2), where one possible reference position is selected and retained in a dedicated spatial working memory. The successful completion of selecting and storing a reference position is detected by mechanisms of neural process control, which are shown in the top left of Fig. 2. Upon the detection, these mechanisms initiate the next processing step, which consists of a visual search process similar to the previous one, but focused on items in target color (here, green). This step involves bringing all items in target color above threshold in the perceptual field through feature attention (the model state in Fig. 2 shows this point of the grounding process). Note that the neurally enforced sequentiality of reference and target selection is mandatory to ensure that reference and target positions are relayed to the correct downstream substrates in the component for relational processing (for details, see Richter, Lins, Schneegans, Sandamirskaya, & Schöner, 2014a, b; Richter et al., 2017). The potential target item positions are then spatially transformed within the relational component, bringing them into a space that is centered on the stored reference position. An activation template, driven by the spatial phrase representation, instantiates the semantics of the phrase’s spatial term within that space and all potential target positions are matched against the template. By this, the best-fitting item position is selected and ultimately projected back into the perceptual field, so that a peak at the target location forms there. At this point, all items in the spatial phrase have been found and neurally instantiated; the phrase has been successfully grounded.

**Fig. 3:**
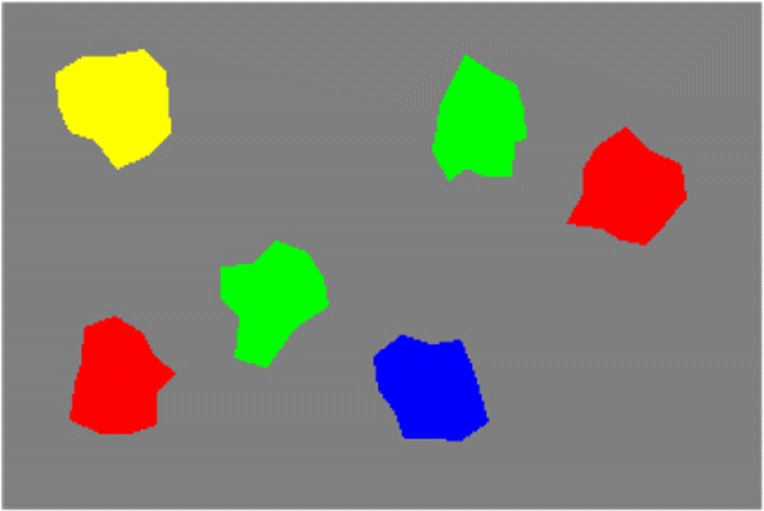
Another scene used as input to the model of spatial language grounding

When grounding the same spatial phrase in a scene such as Fig. 3, which contains two items that share the reference color (red), the model may initially select the incorrect reference item, as it is not known at the time of the selection which of the items in reference color the phrase refers to. In this case, the lack of appropriate target items is detected by mechanisms of neural process control (Richter et al., 2012; Sandamirskaya & Schöner, 2010) and additional grounding attempts occur in sequence until the correct item pair is found.

In summary, the model autonomously realizes the essential steps postulated by Logan and Sadler (1996), including the capability to sequentially test different hypotheses about possible referents of a spatial phrase (similar to Logan, 1994).

**Fig. 4:**
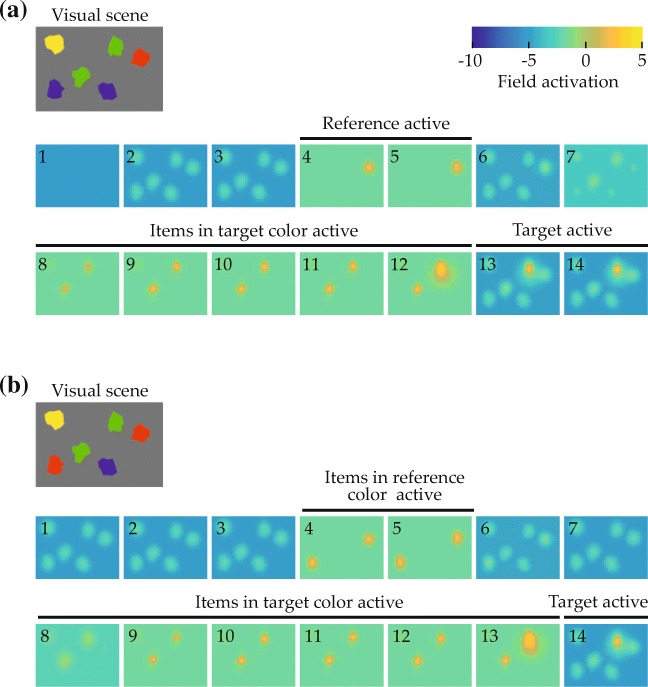
Evolution of activation in the perceptual field during grounding the spatial phrase “The green item to the left of the red item” in the depicted visual scenes. Activation snapshots are numbered in temporal order and show maximum activation along the perceptual field’s color dimension. *Labeled black bars* indicate periods where output is produced at the indicated items’ locations

The model is constrained by neural principles articulated in DFT (Schneegans et al., 2015a), by evidence for sequentiality in relational processing, and by capacity limitations in attentional function (both discussed in the previous section; e.g., Franconeri et al., 2012; Franconeri et al., 2009; Logan, 1994; Treisman & Gelade, 1980), all broadly consistent with a theoretical account in DFT of visual feature representation and feature binding (Schneegans, 2016; Schneegans et al., 2015b). These constraints lead to the hypothesis that grounding always entails the attentional selection of, first, all potential reference items and, subsequently, all potential target items. Importantly, the model postulates that this involves activating all items of the matching color at a point during the selection process. This becomes visible in the evolution of activation in the perceptual field during grounding, which is shown for the two example scenes in Fig. 4: Peaks of activation arise at all locations where items in reference color are located, and the same is true for items in target color.

Due to its unique role of linking space to item features (here, color), the perceptual field serves as a hub that connects multiple neural systems. Conceptually similar fields are implicated in various functions such as visual working memory and change detection (Schneegans et al., 2015b; Schneegans 2016; Zibner et al., 2011, 2017) as well as driving motor systems (Tekülve et al., 2016; Zibner et al., 2015). We postulate that activation peaks in the perceptual field (or a related neural representation) may impact motor planning (Cisek & Pastor-Bernier, 2014; Cisek, 2007; Cisek & Kalaska, 2005; Bastian et al., 1998).

We thus expect that the attentional selection of all potential referents of the spatial phrase may lead to motor signatures during a grounding task. This includes all items sharing the target or the reference color. If motor signatures arise from the activation of potential reference items, rather than only from the activation of potential target items, this shows that motor planning is susceptible to influences from higher cognitive processes during the grounding of relations.

#### Experiments

We conducted four experiments that probe how spatial language grounding is tied to the neural representations of visual space and associated motor responses. The experimental paradigm closely resembled the grounding scenario solved by the model described above. Participants read a spatial phrase which described a relation between two colored items, such as “The green item to the left of the red one.”, and then saw a visual scene composed of 12 colored items, including the described pair. A cursor controlled via the computer mouse had to be moved from a starting point to the target item of the phrase (green in the example) while the cursor trajectory was recorded. By definition, the target item had the target-defining color mentioned in the phrase and at the same time matched the spatial term better than any other item in that color. Other items in the scene included a reference item (red in the example), relative to which the spatial term must be applied in order to find the target; one or more distractor items, defined by sharing the target color but providing a quantitatively worse match to the spatial term; and, in some experiments, items that shared the color of the reference item but were not combined with a target item to form the relational pair described in the phrase. The remaining items were differently colored fillers.

The mouse trajectories were examined for biases toward items that according to the model must be brought into the attentional foreground in the grounding process. This included distractors and items in reference color, as these could potentially take on the roles contained in the spatial phrase. Only six clearly distinguishable colors were used in each display, making visual search among the items highly efficient (Wolfe & Horowitz, 2004; Wolfe et al., 1990). It was therefore assumed that items would be identified rapidly as candidates or non-candidates for the different roles, which entails ruling out reference and filler items as potential movement goals at an early stage of the grounding process. Attraction to items in reference color was therefore of particular interest, since these items gained relevance only from their computational role in the grounding process whereas it could be determined rapidly through visual search that they did not pose potential movement goals. Filler items were not expected to impact the grounding process systematically, again due to the ease with which relevant items can be singled out through visual search based on color. This expectation is also supported by a study in which the impeding effect of distracting items irrelevant to a sought relation disappeared when target and reference were colored differently from the other items (Logan & Compton, 1996).

Experiment 1 looked for attraction toward a uniquely colored reference item and for attraction toward a distractor item. Experiment 2 served to disambiguate the nature of two effects observed in Experiment 1, namely those of the reference item and of the spatial term used in the phrase. This involved changing the directionality of response movements from vertical to horizontal. Experiment 2 thus also generalized the findings of Experiment 1 to this different response metric. Experiment 3 further tested the hypothesis that effects observed in Experiments 1 and 2 were signatures of grounding processes rather than based merely on the fact that the colors of distractor and reference item were mentioned in the phrase. For this, it was probed whether attraction caused by a competing relational pair, composed of a distractor and an additional item in reference color, transcended the sum of biases evoked by individual items in reference or target color that were not part of such a pair. Experiment 4 sought to provide further support for the interpretation that in Experiment 3 additional attraction had been caused by the combination of items into a relational pair rather than by a generic interaction between closely spaced items in task-relevant colors. This was done by comparing attraction toward a competing relational pair to attraction caused by an analogous pair in which both items shared a single task-relevant color.

Key aspects of the cognitive task used in these experiments differed from previous mouse tracking work, which required some adjustments in the employed methods. Most importantly, the space in which cognitive processes operated to solve the task was congruent with the response space, and this space was structured in a complex and variable manner. As described, mouse tracking research has instead focused on abstract cognitive tasks, and typically considered only a single, spatially fixed source of potential attraction, usually the sole alternative response option, so that any deviation could be interpreted in relation to that source (but see Scherbaum et al. 2015). Here, each visual display contained multiple effect sources, whose locations varied from trial to trial, and who could be situated on either side of the straight path to the ultimate movement target (relative to which trajectory deviation was measured). Biases induced by these sources were expected to superimpose in each trajectory and, due to the variable placement, to do so in a different manner in each trial. In effect, net trajectory biases could potentially go in either direction and even change directionality over movement time. Measuring the effects of individual sources thus required a systematic yet flexible manner to generate the complex visual displays, combined with specific measures to counterbalance the impact of confounding influences for analysis.

## General methods

Aspects common to all experiments are described here. Specific aspects will be covered in the experimental sections.

### Participants

Participants were recruited separately for each of the four experiments, by notices around the local campus. They signed informed consent and received monetary compensation for participation. The participants were naïve to the experimental hypotheses, native German speakers, had self-reported normal or corrected-to-normal vision, and no color vision deficiencies.

### Apparatus and stimuli

The experiments were implemented and run using MATLAB R2017a and the Psychophysics Toolbox 3 (Brainard, 1997; Pelli, 1997; Kleiner et al., 2007), and presented on a 22” LCD screen (Samsung, 226BW at 1920 × 1080 resolution; size of visible image 475 mm× 297 mm) at a viewing distance of approximately 70 cm (thus subtending approximately 40.4^∘^× 22.99^∘^ visual angle, v.a.). Trajectories were collected using a standard computer mouse (Logitech, M-UAE96, approximate sampling rate 92 Hz; Experiments 2 to 4 instead used a Roccat Kone Pure mouse, effectively sampling at approximately 400 Hz). Mouse cursor speed was set such that mouse movement on the tabletop translated to cursor movement over the same physical distance on the screen, to make motions more similar to natural arm movements and simplify cognitive transformation from hand coordinates to screen space (see, e.g., Krakauer, Pine, Ghilardi, & Ghez, 2000).

#### Spatial phrases

Spatial phrases were in German and followed the scheme *article – target – spatial term – reference*, as in the example “Das Grüne rechts vom Roten.”, which translates to “The green [one] to the right of [the] red [one]”. The *article* was always “Das”, the *target* was taken from the set {Rote, Grüne, Blaue, Gelbe, Weiße, Schwarze}, the *reference* from the set {Roten, Grünen, Blauen, Gelben, Weißen, Schwarzen}, translating to “the {red, green, blue, yellow, white, black} one”, and the *spatial term* was taken from the set {links vom, rechts vom, über dem, unter dem}, translating to {left of, right of, above, below}.

The spatial phrases thus denoted a target item by a combination of a color (“green” in the above example) and a position given relative to a reference item (“right of”), which was specified only by its color (“red”). Which of the six colors took the role of target and reference was determined randomly for each trial. The relational description provided by the spatial phrase was qualitatively valid for at least one visual item in the associated visual scene.

#### Visual scenes

Figure 6 shows an annotated example display from Experiment 1, illustrating the general structure of the visual scenes (only the start marker and the visual items were visible to the participants). The visual items were irregular polygons, generated randomly for each trial and having a diameter between 8.2 and 16.4 mm (0.67 and 1.34^∘^ v.a.; circles in Fig. 6). They could be colored green, red, blue, yellow, black, or white, were constrained to a rectangular stimulus region (see Fig. 6), and retained a minimum border-to-border distance of 0.5 mm.

**Fig. 5:**
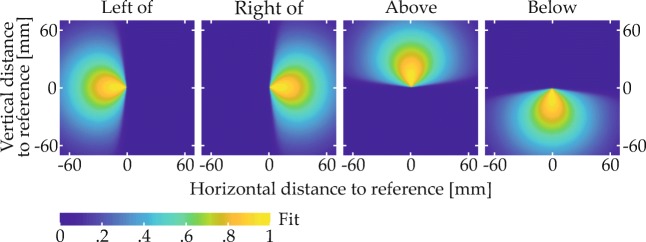
Fit functions describing the quantitative match to different spatial terms in relation to a reference item at the origin

**Fig. 6:**
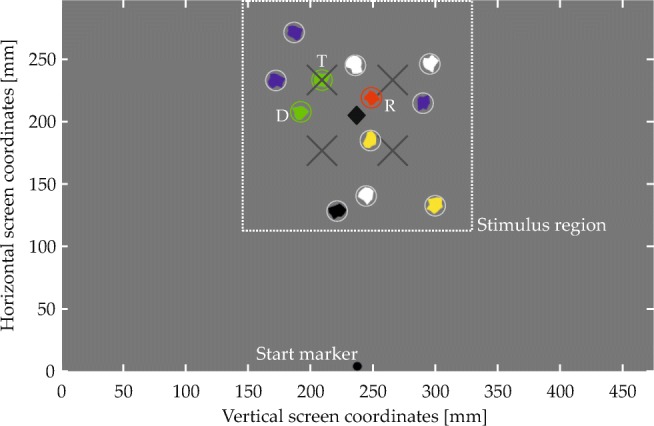
Display configuration in Experiment 1. The item array corresponds to the spatial phrase “The green item to the left of the red item”. T denotes the target, D the distractor, and R the reference item. See text for details

A subset of items in each scene matched one of the two colors named in the spatial phrase and were thus expected to give rise to behavioral effects. These items’ spatial arrangement was determined in a controlled manner. Most importantly, every scene contained a target item and a reference item. The spatial arrangement of these items relative to each other was determined with the help of two-dimensional fit functions, which described for each spatial term how well different spatial coordinates, defined in relation to the reference location at the origin of the coordinate space, matched the term’s semantics (Fig. 5; see Appendix A for the underlying equation). To ensure that targets matched the spatial term well, they were placed in a region of the fit function where fit exceeded a threshold value (given in the experimental sections; see Fig. 9a for an example). To sample the space in this region approximately uniformly, possible target positions were located on the junctions of an equally spaced square grid superimposed on the region (see Fig. 9b for an example). One of the resulting positions was selected for each trial, thereby fixing the relative positioning of reference and target. The placement of the resulting two-item configuration within the final display was then determined such that the target item was positioned in one of four possible target locations (gray X’s in Fig. 6). Each of the four possible target locations was used with each possible target-reference configuration, meaning that each target-reference configuration was used in four visual scenes.

Other items sharing colors from the spatial phrase were present only in some experiments and conditions. This included distractor items and, for some scenes in Experiments 3 and 4, a pair of items that instantiated the inverse of the relation described in the phrase, or items sharing the reference color without being part of such a pair. How these items were placed is described in the experimental sections.

Finally, after fixing the positions of the above items, filler items (encircled gray in Fig. 6) were added to arrive at a total of 12 visual items in each scene. This made the arrays more similar to real-world scenes that naturally afford the use of spatial language to point out a specific item, rather than denoting a target based on simple features or based on the overall gestalt of the array. The fillers were randomly placed in the stimulus region and their colors were randomly taken from the colors not mentioned in the spatial phrase (i.e., from a pool of four colors). A constraint on filler placement was that the center of mass across all items in a scene (black diamond in Fig. 6) had to be congruent with the center of the stimulus region (with a tolerance of ± 0.8 mm in either direction for technical reasons). This means that the center of mass always was in the horizontal screen center (or vertical, in Experiment 2), which simplified counterbalancing potential biases toward either of the two as later described. This constraint also made the average position of the item array independent of the target location, which prevented participants from inferring the approximate location of the target item on that basis.

### Procedure

**Fig. 7:**
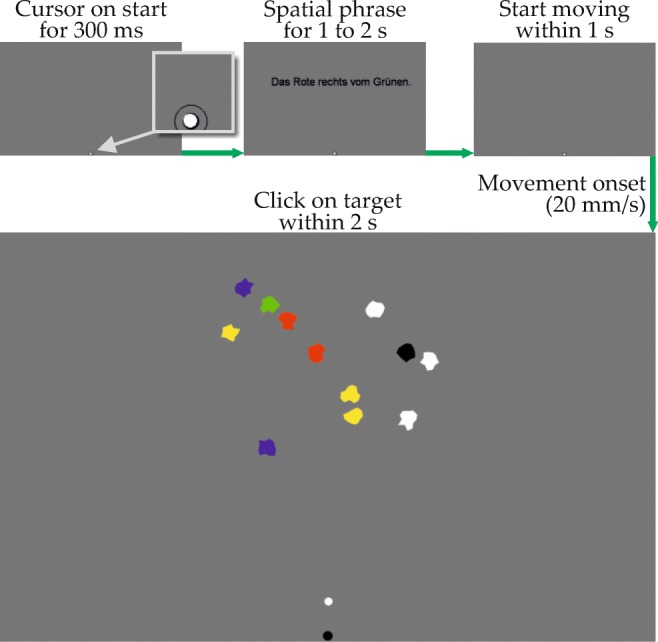
A single trial. Note that the spatial phrase is not drawn to scale

The procedure is illustrated in Fig. 7. To start a trial, participants moved the mouse cursor, a white dot, onto a black start marker centered in the bottom of an otherwise gray screen. After resting there for 300 ms, the spatial phrase appeared at a position somewhat random around the center of the stimulus region (± 48 mm/20 mm in horizontal/vertical direction; text was in Arial and 8.8 mm high). The phrase was visible for a random duration between 1 and 2 s to counteract anticipatory responses. Phrase offset was marked by an auditory beep. Participants were instructed to start movement in upward direction (or rightward, in Experiment 2) within 1 s after the phrase had disappeared. Movement onset was defined as cursor movement faster than 20 mm/s, which was assessed by continuously monitoring traveled pixels within 20ms sampling intervals (as described above, physical mouse movement distance was equivalent to physical cursor movement distance, so that the threshold as well applied to both).

If mouse movement occurred too early or too late, the trial was aborted with appropriate feedback and presented again at a later point. Importantly, the array of visual items appeared only upon movement onset, in order to force selection of the motor goal into the same time window as attentional selection processes associated with the grounding task. Also, it has been shown that presenting stimuli only after movement onset produces more consistent deviation than showing stimuli first (Scherbaum & Kieslich, 2017).

The participant’s task was to select the item which in his or her opinion best matched the preceding phrase (participants could select any item). Starting from movement onset, participants had 2 s to select an item by clicking it (any mouse click closer to an item’s center than the maximum item radius of 8.2 mm was registered as selection of that item). If no selection occurred in that time window, the trial was aborted with appropriate feedback and presented again at a later point. The time limit served to prevent participants from stopping mouse movement while grounding the relation, so as to time-lock movement onset and the start of relation grounding. The allowed duration was based on pilot work and adjusted to impose a sense of time pressure without requiring hasty responses. Trials exceeding the time limit mainly occurred during the first few trials, before participants fully adapted to the paradigm. After item selection, the next trial followed.

Apart from the instruction to select the best-matching item, participants were told that there were no correct or incorrect responses (in particular, they were not made aware of the technical distinction between targets and distractors) and that the items did not pose obstacles for mouse movement. Prior to the experimental trials, the experimenter demonstrated the procedure by completing two trials (once choosing the distractor and once choosing the target) and each participant completed 13 practice trials with no time constraints.

### Analysis

Only data from trials with correct responses entered analysis. Responses counted as correct if participants had selected the item that fitted the spatial phrase best according to the fit function (i.e., the target item). Furthermore, trajectories with sharp turns were excluded from analysis. This derives from previous mouse tracking research in which distributions of curvature have been used to determine whether responses might stem from two distinct populations of trials, one where an initial response decision is corrected mid-flight (leading to high deviation) and another where the initial decision remains unchanged so that trajectories are affected only by graded influences from other sources (leading to low deviation; Farmer, Anderson, & Spivey, 2007; Freeman & Dale, 2013; Hehman et al., 2015).

Due to the specifics of the current paradigm, we had to assess curvature in a different manner than previous mouse tracking studies, which have typically used area under the curve or maximum deviation (Freeman & Dale, 2013; Hehman et al., 2015). These latter methods measure curvature as deviation from the direct path aggregated over movement time and as the largest observed distance from the direct path, respectively. They thus express the *global* degree of curvature in a trajectory, which is useful when deviation is expected to occur only in one particular direction, for instance, toward the nonselected alternative out of two response buttons. In the current experimental setup, it was expected that multiple potentially opposing biases would jointly affect individual trajectories. For instance, a trajectory’s shape may be codetermined by attraction toward a distractor item located to the left of the direct path and by attraction toward a reference item located to the right of the direct path. With superimposed opposing biases, global measures of curvature can yield misleading values. In the above case, for instance, maximum deviation would capture only the larger of the two opposing biases and any global measure of curvature may be erroneously reduced by the influence of the counteracting bias. Thus, global measures are difficult to interpret with mixed-direction biases.

To circumvent these problems, we sought to identify possible redecisions mid-flight using a measure of *local* curvature that yields high values at abrupt turns but is unaffected by the trajectory distance from the direct path. It was computed by an algorithm (described in detail in Appendix B) which for regularly spaced points along the length of trajectories yields curvature values between zero (straight line) and *π* radians (antiparallel trajectory segments). To identify trials where an abrupt redecision or a similar local event may have taken place, the maximum curvature value within each trajectory was determined and compared to a fixed threshold value of 0.933 radians. Trajectories exceeding this threshold were excluded from analyses. Apart from cleaning the data set of possible redecisions, this also served to exclude outlier trials with extreme deviations that hinted at momentary failures to coordinate mouse movement and subsequent corrections of movement direction.

The outcome of the exclusion procedure was governed by three parameters: the threshold value and two parameters of the curvature computation algorithm itself (the latter two are explained in Appendix B). The three parameters were tuned based on the trajectory data set of Experiment 1 and the obtained values were used for all trajectories and experiments. The tuning procedure involved plotting excluded and included trajectories for different parameter sets and manipulating parameters until a balance was found of reliably excluding apparent redecisions and outliers without discarding overly large portions of the data. For instance, the algorithm was tuned to retain trajectories with very brief deviations that appeared to result from slightly overshooting the target or from minor imprecisions in mouse handling. Since, regardless of the measure of curvature used, no objective criterion is known that would perfectly distinguish trials with redecisions from those where only graded attraction is present, a certain degree of subjective judgment was necessarily involved in the choice of parameters. To provide an impression of retained and excluded trajectories obtained with our parameters, Appendix B shows some examples from these two sets. We further sought to alleviate this issue by statistically examining the distributions of maximum curvature across all trials for signs of distinct response populations (i.e., bimodality; described in detail under statistical methods). This analysis was conducted once including and once excluding those trajectories that exceeded the curvature threshold, in order to test for the presence of different response populations in general as well as in the cleaned data set used in the main analyses.

#### Trajectory preparation

**Fig. 8:**
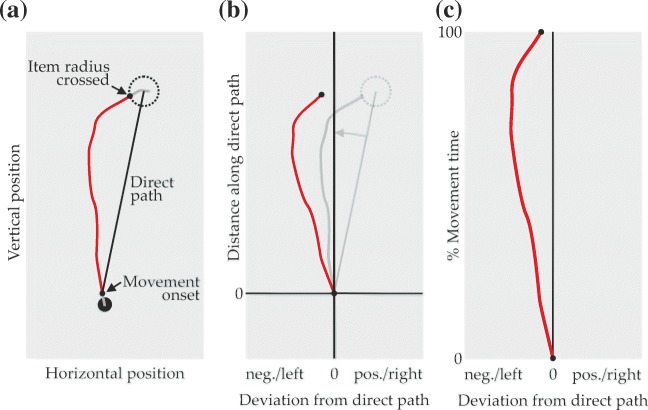
Trajectory preparation steps. **a** Trajectory portions before movement onset and after crossing the item radius (*gray*) were removed for analysis. **b** Translation and rotation to make the direct path congruent with the *y*-axis. **c** Normalization over movement time. The *red trajectory* is hypothetical data

Trajectories were trimmed to start with the first data point after movement onset and to end with the last data point before crossing an 8.2mm radius around the selected item’s center (equaling the maximum possible item radius; Fig. 8a). Trimmed trajectories were translated to place the first data point at [0,0] and then rotated around that point such that the center of the selected item would be placed on the positive *y*-axis (i.e., at *x* = 0; Fig. 8b). This entailed that final trajectory points tended to lie not at *x* = 0, but somewhat lateral to the *y*-axis, depending on where the radial border of the selected item had been crossed, so that any deviations affecting trajectories until the end of the movement were retained in the rotated versions.

Through these transformations, the direct path (see Fig. 8a), defined as the straight line from the point of movement onset to the center of the selected item, was made congruent with the *y*-axis. Thus, as shown in Fig. 8b, x-coordinates in the transformed trajectories are equivalent to deviation from the direct path, with negative values corresponding to biases to the left of the direct path and positive values representing biases to the right (sides are given relative to the “direction of travel” toward the target). To enable averaging, deviation data was time-normalized (Fig. 8c) by linearly interpolating x-coordinates over 151 equally spaced steps of movement time. The data points of averaged trajectories thus combine deviation data from the same proportion of time elapsed since movement onset.

#### Examining trajectory biases

We were interested in whether movement trajectories were attracted by visual items whose colors were mentioned in the spatial phrase. To examine this, mean deviation over time was compared between conditions in which an item of interest was located to the left or to the right of the direct path, respectively. Depending on the effect under scrutiny, the item of interest was either a distractor item or the reference item. In Experiments 3 and 4, it could also be a pair of closely spaced items (which was treated as a single ”item” of interest for matters of analysis) or an item sharing the reference color.

To fully isolate the effect of the item of interest in a given comparison, the impact of several interfering influences needed to be counterbalanced. First, we expected all items that shared a color from the spatial phrase to codetermine trajectory shape in every trial. Second, a trajectory bias toward the screen center was expected, since participants were instructed to start movement into an upward direction (or rightward, in Experiment 2) before the visual items appeared. Third, a bias toward the center of mass across items was expected in early trajectory portions, as participants may have deemed all items potential targets during the short time window preceding color-based visual search. Fourth, we conjectured that spatial terms might impact trajectory shape in a systematic manner, as suggested by priming evidence (Tower-Richardi et al., 2012).

To balance out these influences, mean trajectories for each participant were composed as averages over several experimental sub-conditions. Each sub-condition included all trials exhibiting a specific combination of spatial term (left, right, above, below), target position (top left, top right, bottom left, bottom right), reference side (left or right), and distractor side (left or right; distractor side was replaced by pair side in Experiments 3 and 4). Trajectories within each sub-condition were averaged, yielding one mean trajectory per sub-condition. The obtained means were then grouped into two sets based on the side of the item of interest for the comparison at hand, and overall means were computed within each of the two sets. The two overall means thus differed only with respect to the side of the item of interest, while each combination of biasing influences was weighted to an equal degree in the final means, regardless of the number of trials in the different sub-conditions. Note that using target position as a factor in defining the sub-conditions took care of balancing out the bias toward the screen center, since the screen center was to the left of the direct path for two of the four possible target positions and to the right of the direct path for the other two. It moreover balanced out a possible bias to the center of mass across items, since the center of mass was made congruent with the screen center during scene creation (see above).

Computing overall mean trajectories from sub-condition means instead of averaging directly over all cases was necessary since case numbers in each sub-condition were partly different. One reason was loss of cases due to incorrect responses (i.e., not selecting the target item) and exclusion of sharply curved trajectories. Furthermore, the algorithm for scene creation produced certain trial types somewhat more frequently than others. For instance, in “left of” trials with target positions on the right side of the display, distractors were slightly more often located to the left of the right-leaning direct paths, since for most target-reference configurations the distractor region covered more space to the left of the target item. The approach we took prioritizes the approximately uniform sampling of possible target and distractor positions over equal trial numbers in the sub-conditions.

A limitation on balancing was that spatial terms could not be fully counterbalanced in those comparisons by reference side where the spatial term axis was orthogonal to the direct paths. We refer to “above” and “below” as having a vertical axis, insofar that these terms’ semantics presuppose a vertical displacement between the reference and the target item. Analogously, we refer to “left” and “right” as having a horizontal axis. The direct paths, on the other hand, were roughly vertical when the start marker was below the item arrays (Experiments 1, 3, and 4) and roughly horizontal when the start marker was to the left of the item arrays (Experiment 2). In trials where the direct path and the spatial term axis were roughly orthogonal to each other, the spatial term prescribed on which side of the direct path the reference item had to be located, because the target item had to match the spatial term. For instance, given a vertical direct path and the spatial term “left”, the reference item must be placed to the right of the direct path in order for the spatial term to hold. This coupling of spatial terms and reference sides entails that any effects of spatial terms and reference item placement will be confounded in the respective comparisons. This will be highlighted when discussing the affected results.

##### Statistical methods

Trajectory data were subjected to repeated-measures analyses in the form of paired-samples *t* tests (all experiments) and repeated measures analyses of variance (ANOVAs; Experiment 3). In both cases, separate tests were performed for the data at each of the 151 interpolated points.

The large number of tests gives rise to the question how many significant results in direct succession correspond to overall significance of the difference between the compared series of data points. Due to the strong interdependence of successive data points in natural movement trajectories (Dale et al., 2007), traditional methods such as Bonferroni correction are not applicable. One view on this matter holds that sequences of statistical tests over movement trajectories should be considered as units that stand for a single comparison of whole trajectories, rejecting the need for alpha correction as long as the outcome of the comparison is presented and interpreted in its entirety (Gallivan & Chapman, 2014; Chapman, 2011). Many researchers in mouse tracking (e.g., Anderson et al., 2013; Bartolotti & Marian, 2012; Duran, Dale, & McNamara, 2010; Freeman et al., 2008; Scherbaum et al., 2015) have instead adopted a bootstrap approach (Efron & Tibshirani, 1993) first introduced by Dale et al., (2007). The method preserves the dependency between time steps and yields an empirical distribution of bootstrap replications over the maximum length of significant sequences. Based on a prespecified *p* value, a criterion for sequence length in the real data is derived beyond which the presence of an overall effect is assumed. In keeping with much of the mouse tracking literature, we adopted this approach as an additional indicator for the overall significance of sustained trajectory deviations. The method was implemented according to the description provided by Dale et al., (2007; see also, Scherbaum et al., 2015). For each comparison we report, a separate criterion was computed based on 10,000 bootstrap replications of maximum sequence length, using the compared data as input to the bootstrap. The derived length criteria required for overall significance were based on *p* < 0.01. In the case of ANOVAs, a separate criterion was obtained for each main effect and interaction.

Distributions of maximum curvature values were examined for signs of bimodality. This was done over all correct trials, including those excluded from the other analyses due to exceeding the curvature threshold and, if bimodality was observed in this full sample, also for the smaller set of trajectories with sharply curved ones excluded. We thereby sought to determine, first, whether two distinct populations of trials were at all discernible and, second, whether trials from both of these populations may still have affected the ultimately analyzed set of trajectories. Bimodality was assessed using Hartigan’s dip test (Hartigan and Hartigan, 1985; Hartigan, 1985) in the MATLAB implementation by Mechler (2002), testing the null hypothesis of unimodality against the alternative hypothesis of multimodality, with *p* values below 0.05 indicating bimodality. We used the dip test instead of the more widely used bimodality coefficient (SAS Institute, 2012) since the distributions of maximum curvature were skewed, which may lead to erroneous detection of bimodality by the bimodality coefficient (Pfister et al., 2013).

Finally, movement times were analyzed in an exploratory manner by comparing them between conditions in a way similar to the trajectories; details are provided in the experimental sections.

## Experiment 1

The first experiment^1^ tested whether attentional selection of a uniquely colored reference item and a distractor item during spatial language grounding affected the shape of mouse trajectories to the target. The target and the distractor were viewed as potential movement goals that must be disambiguated through grounding the spatial phrase. The distractor was therefore hypothesized to metrically attract the trajectories. The unique reference item was expected to be ruled out as a potential movement goal by the participants early on but was still expected to be attentionally selected in the grounding process due to its computational relevance. The reference item was therefore as well hypothesized to attract mouse trajectories.

### Methods

#### Participants

The 12 participants (five female, seven male) were 27.4 years (SD = 3.8 years) old on average and received €10 for participation.

#### Visual scenes

**Fig. 9:**
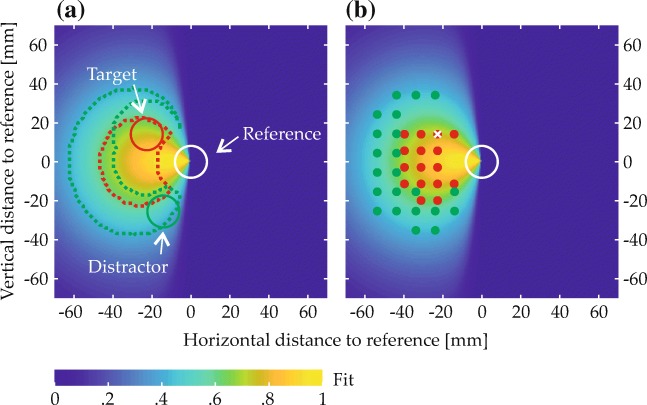
**a** Regions eligible for target and distractor placement (*red and green dotted lines*, respectively) and one possible placement of target and distractor. **b** All possible target positions (*red*) and all possible distractor positions (*green*) for the target position that is marked with a *cross*. *Circles* illustrate the approximate item extent (maximum radius) and *dots* mark item centers

An annotated example display for Experiment 1 is shown in Fig. 6. Possible target positions in the visual scenes were located in a region of the fit function where fit was higher than 0.6, illustrated by the dotted red outline in Fig. 9a (only the fit function for the spatial term “left” is shown; scene creation and the resulting item positions were analogous for the other spatial terms). The spacing of the grid for target placement within that region was adjusted to obtain 16 possible target positions (red dots in Fig. 9b).

A set of possible distractor positions was created separately for each of the 16 target positions. In each case, distractors could be placed in a region of the fit function where fit was higher than 0.4 and at least 0.03 lower than the fit value of the target position at hand (e.g., the green outline in Fig. 9a shows the distractor region for the annotated target and one possible distractor placement; in Fig. 9b green dots indicate all possible distractor positions for the target position marked with a white cross). Out of the resulting distractor positions one was used per trial, paired with the respective target position. Due to the dependency of the distractor region on target fit and position, its shape and size was different for each target position. In consequence, the number of distractor positions varied from 16 to 25 (mean 20.9) between target positions.

In a random subset of scenes (27%) one filler was given the same color as the target and the distractor, as an additional incentive to evaluate the spatial relation. The respective filler had to be located on the side of the reference item opposite to that denoted by the spatial term, so that it did not pose a qualitative match to the term, and it had to be separated from the reference item along the term’s axis (e.g., horizontally for “right”) by at least 28.3 mm (2.32^∘^ v.a.). The effect of this item was not specifically analyzed, but cursory analysis of the data without the trials including such an item showed that results were not markedly changed.

Together, there were 335 different configurations of target, reference, and distractor items for each of the four spatial terms. Each of these was used with each of the four possible target locations, leading to a total of 5360 trials. The trials were randomly assigned to the participants, so that each participant completed 446 trials and one completed eight more to use the entire trial set.

#### Analysis

Trajectory deviation from the direct path as a function of the elapsed proportion of movement time was used as the main dependent measure. Analyses focused on the factors *reference side* and *distractor side*, each with the two levels *left* and *right* (of the direct path). To assess the effect of reference side, three planned contrasts compared mean deviation between left and right conditions. One compared trajectories across spatial terms, one included only horizontal-axis spatial terms (“left” and “right”), and one included only vertical-axis spatial terms (“above” and “below”). The effect of distractor side was assessed with three analogous contrasts. Inflated type I error risk over these comparisons was addressed by choosing *p* < 0.01 for each *t* test.^2^

Paired-samples *t* tests were used to compare movement times between distractor sides, between reference sides, and between spatial term axes. Also, movement time difference scores between distractor sides and between reference sides were compared between spatial term axes (i.e., between horizontal axis trials with the terms “left” or “right” and the vertical axis trials with the terms “above” or “below”). Due to the exploratory nature of these comparisons, each test used *p* < 0.05.

### Results

When asked, participants reported not to have noticed that possible target positions were restricted to four screen locations (some noted that targets tended to be located around the center area of the item arrays rather than in the outer regions). Movement onset was generally registered close to the center of the start marker (M = 2.14 mm, SD = 1.97 mm).

**Fig. 10:**
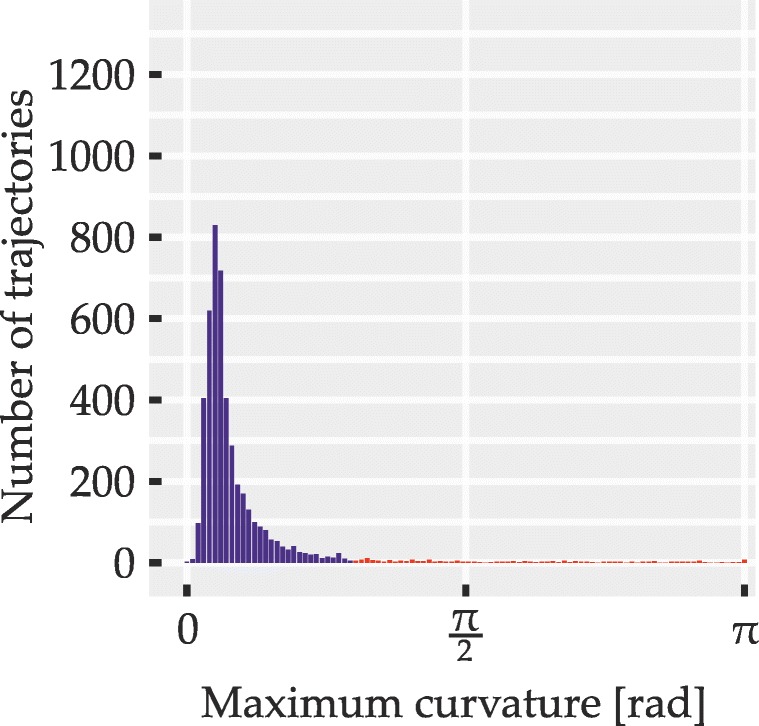
Distribution of trajectories over maximum curvature values in Experiment 1. *Red bars* correspond to trajectories that were discarded due to high curvature. Only correct responses are shown

A total of 5245 trajectories was obtained (115 were lost due to technical issues). Of these, 5003 (95.39%) were below curvature threshold (M = 416.92, SD = 35.91 equaling M = 95.3*%*, SD = 2.76*%*). Of the non-curved trajectories, 90.17% (4511) were correct responses and thus entered further analysis (86.01% of all obtained trajectories). Participants achieved a mean accuracy of 90.18% (SD = 3.34*%*) and their mean movement time was 1073 ms (SD = 112 ms). The above numbers are based on simple averaging over the respective trial ensembles; mean data reported from here on is based on balanced means as described. Figure 10 shows the empirical distribution over maximum curvature values for all correct responses, with red bars indicating curvature above threshold (i.e., trajectories excluded from other analyses). For the distribution, Hartigan’s dip test indicated no bimodality (*p* > 0.05).

**Fig. 11:**
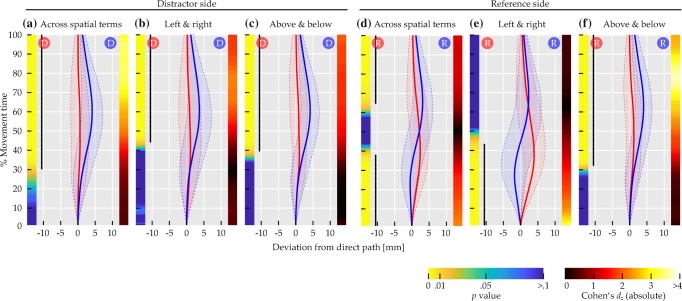
Comparisons of mean deviation for Experiment 1. *Solid red and blue lines* show mean trajectory data, with *red and blue circles* labeled ‘D’ or ‘R’ in the top of the panels indicating distractor or reference side for the correspondingly colored trajectory. *Transparent regions delimited by dashed lines* indicate between-subjects standard deviation. Left color maps indicate *p* values at that time step, right ones indicate effect sizes. *Black dotted lines on the left* span time steps where differences were significant (*p* < 0.01)

The left side of Fig. 11 visualizes the results of comparisons by distractor side, where red and blue circles labeled ‘D’ in the top of each panel indicate distractor side for the correspondingly colored mean trajectory.

Across spatial terms, trajectories diverged in a way consistent with a bias toward the distractor (Fig. 11a), with 106 successive time steps showing significant differences at *p* < 0.01, thus exceeding the bootstrap criterion (*p* < 0.01) of 18 time steps. The sequence of significant differences extended from 30.46 to 100% of movement time. For horizontal axis spatial terms, the bias toward the distractor was present as well (Fig. 11b), with 85 successive time steps showing significant differences at *p* < 0.01, exceeding the bootstrap criterion (*p* < 0.01) of eight time steps. The sequence of significant differences extended from 44.37 to 100% of movement time. Similarly, for vertical axis spatial terms, the bias toward the distractor was present (Fig. 11c), with 92 successive time steps showing significant differences at *p* < 0.01, exceeding the bootstrap criterion (*p* < 0.01) of 14 time steps. The sequence of significant differences extended from 39.74 to 100% of movement time.

The right side of Fig. 11 visualizes the results of the comparisons by reference side, where red and blue circles labeled ‘R’ in the top of each panel indicate reference side for the correspondingly colored mean trajectory. Across spatial terms, a mixture of two biases was visible (Fig. 11d). In the first half of movement time, trajectories diverged in a way consistent with a bias away from the reference item. This effect spanned 56 successive time steps with significant differences at *p* < 0.01, exceeding the bootstrap criterion (*p* < 0.01) of six time steps. For this effect, the sequence of significant differences extended from 1.32 to 37.75% of movement time. In the second half, trajectories diverged in a way consistent with a bias toward the reference. This effect spanned 54 successive time steps with significant differences at *p* < 0.01, as well exceeding the bootstrap criterion (*p* < 0.01) of six time steps. For this effect, the sequence of significant differences extended from 64.9 to 100% of movement time.

For horizontal axis spatial terms (Fig. 11e), only the early divergence consistent with a bias away from the reference remained. Note that, due to the coupling of reference side and spatial term in trials with horizontal axis spatial terms, this bias is also congruent with movement in the direction described by the spatial term. The divergence was present over 64 successive time steps showing significant differences at *p* < 0.01, exceeding the bootstrap criterion (*p* < 0.01) of 34 time steps. The sequence of significant differences extended from 1.32 to 43.05% of movement time. For vertical axis spatial terms (Fig. 11f), in contrast, only the late divergence consistent with a bias toward the reference remained. The divergence was present over 103 successive time steps showing significant differences at *p* < 0.01, exceeding the bootstrap criterion (*p* < 0.01) of 21 time steps. The sequence of significant differences extended from 32.45 to 100% of movement time.

Condition-specific movement times are listed in Table 1. The *t* tests on movement time data showed no significant impact of distractor side, reference side, or spatial term axis (*ps* > 0.05). Similarly, there was no significant impact of spatial term axis on movement time difference scores between distractor sides or reference sides (*ps* > 0.05).

**Table 1:** Movement times and standard deviations (SD) for Experiment 1

	Distractor side				Reference side				Overall	
	Left		Right		Left		Right			
Spatial terms	Mean	SD	Mean	SD	Mean	SD	Mean	SD	Mean	SD
Overall	1072	113	1075	113	1074	115	1073	110	1073	112
Left/Right	1088	104	1085	114	1092	110	1081	108	1087	108
Above/Below	1065	123	1067	111	1060	119	1072	117	1066	116

### Discussion

In the vast majority of trials, participants selected the target item, suggesting the employed fit functions appropriately captured the spatial terms’ semantics. The majority of trajectories were smoothly curved, showing that motor responses were mostly subject to graded attraction, whereas decisions about motor targets may have been abruptly revised in only very few trials. Together with the absence of bimodality in the curvature distribution this suggests that the motor responses were not governed by fundamentally different processes from trial to trial.

The mouse paths to the target item displayed biases into different directions. Three effects were observed. First, there was a *distractor effect*, which biased trajectories to the side of the direct path on which the distractor item was located. The effect was observed to comparable degrees when the target position relative to the reference item was specified by horizontal axis spatial terms (“left of” or “right of”) and when it was specified by vertical axis spatial terms (“above” or “below”). Its onset occurred after approximately a third of the total movement time. The distractor effect is in line with the notion that target and distractor were viewed as potential movement goals that must be disambiguated through grounding the spatial phrase, paralleling earlier studies where initial uncertainty over the ultimate movement goal was induced through other means such as delayed cuing (e.g., Chapman et al., 2014; Gallivan & Chapman, 2010).

Second, there was a *reference effect*, which consisted of trajectory attraction toward the side of the direct path where the reference item of the spatial phrase was located. In the mean data across spatial term axes, this effect was visible within the last third of movement time. In trials using the spatial terms “above” and “below”, its onset occurred after approximately a third of the total movement time, and the effect was considerably more pronounced, likely due to not being superimposed with an effect of the spatial term, as discussed below. An attraction to the reference item was not observed for horizontal axis spatial terms, which again was probably due to superimposition with an effect of the spatial term in these trials. That the reference effect was weaker in the across-spatial-term comparison is most likely attributable to the mixture of trials from each spatial term axis in that comparison, so that an average of the effect’s presence in vertical axis spatial terms and its absence in horizontal axis spatial terms was observed. Since the reference item could likely be ruled out as a potential movement goal through quick visual search, the reference effect suggests that its impact on trajectories was due to its involvement in the cognitive process of spatial language grounding. Note that if this was the case for the reference item, the same mechanism may have contributed to the distractor effect as well, beyond the distractor’s role as a potential action target.

Third, the *spatial term effect* was a bias with a very early onset, pointing in the direction described by the spatial term. It was visible in the comparisons by reference side and there only in the mean data across spatial terms and, more strongly and somewhat more extended, in trials with horizontal axis spatial terms. In both cases, the spatial term effect occurred immediately after movement onset and remained observable over approximately 40% of the total movement time. The early onset suggests that participants were already moving in a direction congruent with the spatial term before the item array appeared. The effect thus cannot have resulted from the arrangement of visual items and, for instance, pose a repulsion from the reference item. Also, if the latter were the case, the effect should have been observable independent of the spatial term. Moreover, recall that the spatial term did not predict the absolute location of the target or its side in the display, since across trials each of the four target locations was paired an equal number of times with each spatial term. Thus, starting movement in the direction described by the spatial term would have been a less viable strategy to decrease target distance than simply starting movement in an upward direction as instructed. These considerations suggest that the spatial term effect can instead be attributed to the semantics of the spatial term, independent of cognitive strategies or visual stimulation. This interpretation is consistent with prior evidence about a biasing impact of cardinal direction prime words (e.g., “north”) on mouse trajectories (Tower-Richardi et al., 2012). The effect also bears similarities to a motor bias evoked by the directionality implied in sentences that were judged for sensibility (Zwaan et al., 2012) and similar embodiment effects (e.g., Glenberg & Kaschak, 2002).

It is unsurprising that the spatial term effect was observed only in the comparisons between reference sides and only for horizontal axis spatial terms (and less strongly in the across-spatial-term comparison). In trials with horizontal axis spatial terms, the side of the reference item relative to the direct path was coupled to the spatial term: When the reference item was on the left side, the spatial term was “right” and vice versa. Thus, in the comparisons by reference side for horizontal axis spatial terms each set of data included only one spatial term, so that its effect could systematically impact the mean trajectories. By contrast, in trials with vertical axis spatial terms reference sides and spatial terms were not coupled, so that possible biases in spatial term direction could not become visible in the balanced means. Such biases would furthermore have acted approximately parallel to the direct paths, making them unlikely to be observable in the deviation measures. In comparisons by distractor side, on the other hand, reference side was generally balanced in the compared means and thereby also any impact of the spatial terms. Regarding the across-spatial-term comparison by reference side, the lower strength and earlier offset of the spatial term effect likely stemmed from combining trials in which the effect was present with trials where it was absent. Finally, although the spatial term effect was generally observed only in the first half of the movements, we surmise that it in fact influenced trajectories over much of the movement time. This is based on the absence of a reference effect in the comparison by reference side for horizontal axis spatial terms, which at first seems difficult to reconcile with the notion that the reference effect was based on the involvement of the reference item in the grounding process. This notion can be retained, however, by assuming that the spatial term effect cancelled out with the reference effect in the second half of that comparison, so that neither effect became visible there.

Concerning the approximate latency of the observed effects, an unbiased picture can be gathered from those conditions where observed deviations were probably not mixtures of multiple effects; this includes all comparisons by distractor side and the comparison by reference side for vertical axis spatial terms. Apart from the spatial term effect, biases’ onsets occurred at approximately 30 to 40% of total movement time. Given an overall mean movement time of 1073 ms, this corresponds to an absolute temporal separation between scene onset and effect onset of approximately 400 ms (these numbers are deliberately kept vague and must be considered with care, as a possible covariation of absolute movement time and effect magnitude is not taken into account; for instance, if the effect estimate is dominated by trials with long movement times, then the absolute time between display and effect onset may be underestimated). These effect onset times are broadly consistent with the time required for visual search with color targets. For instance, Wolfe et al., (1990) found reaction times of approximately 500–600 ms for detecting a color target among up to 32 items in ten different colors (note that these reaction times include the motor response); search was highly efficient with minimal reaction time slopes over increasing item number. Together, this suggests that items in the scenes used in the experiments here could be distinguished very quickly via efficient visual search. It is thus likely that the observed effects were not affected by difficulties in finding the relevant items among fillers or distinguishing their different roles.

The claim that the mere involvement of visual items in the grounding process causes motor biases hinges on the reference effect. Consolidating the findings of Experiment 1 thus requires showing that the reference effect is indeed universal across spatial terms and not a peculiarity of “above” and “below” or of the specific response metrics afforded by the task. This is further examined in Experiment 2.

## Experiment 2

The main goal of Experiment 2,^3^ was to confirm that the reference effect is universal across spatial terms and response metrics. We conjectured that its absence for horizontal axis spatial terms in Experiment 1 was due to its overlap with the spatial term effect, not due to a complete absence of an impact of the reference item. In addition, Experiment 2 aimed to replicate the distractor effect seen in Experiment 1 using different response metrics.

The paradigm was largely analogous to Experiment 1. The main difference was that responses were made along a roughly horizontal rather than vertical axis, from the start marker on the left side of the screen to targets on the right side of the screen. Compared to Experiment 1, this resulted in a switched relationship between the principal movement direction and the spatial term axes: The axis of the terms “left” and “right” was now approximately parallel to the direct paths and the axis of the spatial terms “above” and “below” was approximately orthogonal to the direct paths. In consequence, the side of the reference item relative to the direct path was now coupled to the vertical axis spatial terms (left side for “below”, right side for “above”) while this coupling was removed for the horizontal axis spatial terms.

It was hypothesized that all effects from Experiment 1 would occur in an analogous manner in Experiment 2, with partly reversed couplings of spatial term axes and effects. The distractor effect was hypothesized to occur equally for both spatial term axes. The spatial term effect was hypothesized to be observable for vertical axis spatial terms but not for horizontal axis spatial terms. Conversely, the reference effect was hypothesized to be observable for horizontal axis spatial terms, but not for vertical axis spatial terms. In other words, the observable signatures of the reference effect and the spatial term effect were expected to be switched between spatial term axes compared to Experiment 1. This rested on the assumption that the two biases would cancel each other out in trajectory portions where both were present, as conjectured based on the results of Experiment 1. This hypothesized pattern of results would argue for the generality of both the reference effect and the spatial term effect over spatial term axes and response axes as well as confirm the robustness of the distractor effect.

### Methods

#### Participants

The 24 participants (15 female, nine male) were 25 years (SD = 4.1 years) old on average and received €10 for participation.

#### Visual scenes

**Fig. 12:**
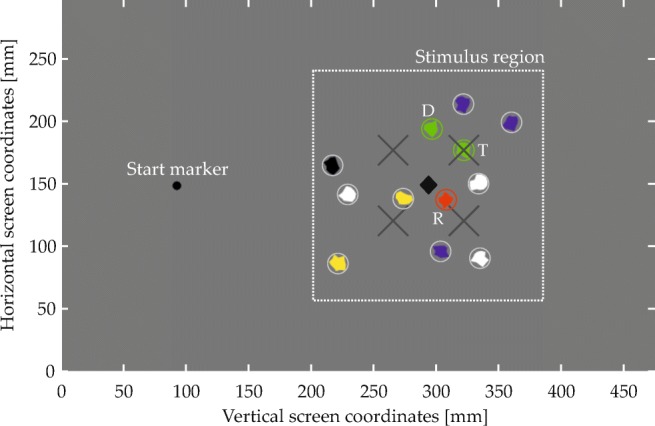
Display configuration in Experiment 2. The item array corresponds to the spatial phrase “The green item above the red item”. T denotes the target, D the distractor, and R the reference item. See text for details

An annotated example display for Experiment 2 is shown in Fig. 12. The arrangement differed from that in Experiment 1 only by clockwise rotation around the screen center by 90 degrees. The extent of the different components and the distances between them were unchanged.

**Fig. 13:**
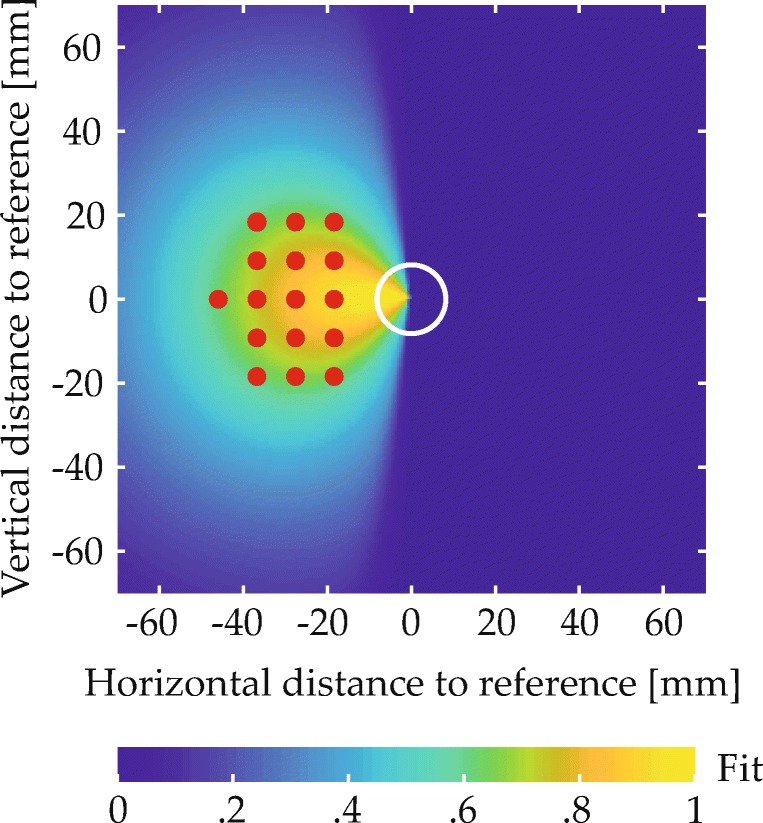
Possible target positions in Experiment 2. *Dots* mark target item centers and the *circle* marks the maximum reference radius

Visual scenes were created by the same methods as for Experiment 1, with the minor change that the placement of the grid of target positions over the fit function’s target region was adjusted such that the possible target positions were distributed symmetrically on either side of the axis of the spatial term at hand (Fig. 13; this was the case for the remaining experiments as well). Due to this, there was a slightly different number of distractor positions for each of the 16 target positions, namely 19 to 24 per target with a mean of 21.8. In turn, this lead to a slightly higher total number of different displays, namely 5584. Since participant number was 24 and thus double the number of that in Experiment 1, the set of trials was doubled as well (i.e., each visual display was presented twice) and trials from the resulting set of size 11168 were randomly assigned to the participants.

#### Analysis

Analysis and design were the same as in Experiment 1.

### Results

When asked, participants reported not to have noticed that possible target positions were restricted to four screen locations. Movement onset was generally registered close to the center of the start marker (M = 2.15 mm, SD = 3.01 mm).

**Fig. 14:**
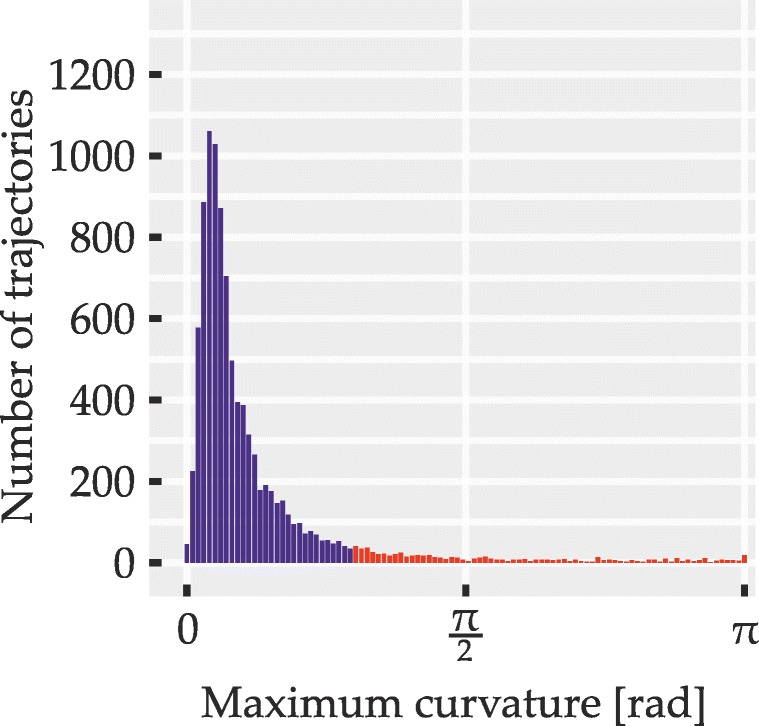
Distribution of trajectories over maximum curvature values in Experiment 2. *Red bars* correspond to trajectories that were discarded due to high curvature. Only correct responses are shown

A total of 11,157 trajectories was obtained (11 were lost due to technical issues). Of these, 10,224 (91.64%) were below curvature threshold (M = 426, SD = 32.9 equaling M = 91.64*%*, SD = 7.09*%*). Of the non-curved trajectories, 86.75% (8869) were correct responses and thus entered further analysis (79.49% of all obtained trajectories). Participants achieved a mean accuracy of 86.59% (SD = 5.68*%*) and their mean movement time was 1057 ms (SD = 129 ms). The above numbers are based on simple averaging over the respective trial ensembles; mean data reported from here on is based on balanced means as described. Figure 14 shows the empirical distribution over maximum curvature values for all correct responses, with red bars indicating curvature above threshold (i.e., trajectories excluded from other analyses). For the distribution, Hartigan’s dip test indicated no bimodality (*p* > 0.05).

**Fig. 15:**
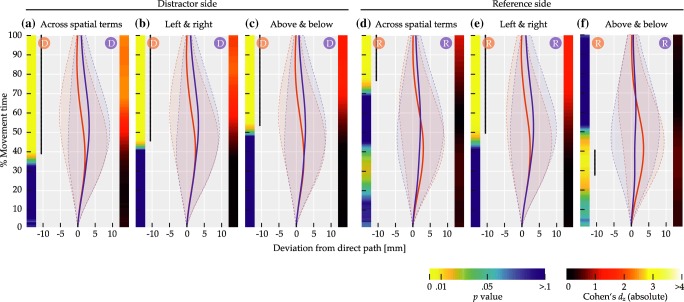
Comparisons of mean deviation for Experiment 2. *Solid red and blue lines* show mean trajectory data, with *red and blue circles* labeled ‘D’ or ‘R’ in the top of the panels indicating distractor or reference side for the correspondingly colored trajectory. *Transparent regions delimited by dashed lines* indicate between-subjects standard deviation. Left color maps indicate *p* values at that time step, right image maps indicate effect sizes. *Black dotted lines* on the left span time steps where differences were significant (*p* < 0.01)

The left side of Fig. 15 visualizes the results of comparisons of mean trajectories by distractor side, where red and blue circles labeled ‘D’ in the top of each panel indicate distractor side for the correspondingly colored mean trajectory.

Across spatial terms, trajectories diverged in a way consistent with a bias toward the distractor (Fig. 15a), with 93 successive time steps showing significant differences at *p* < 0.01, thus exceeding the bootstrap criterion (*p* < 0.01) of seven time steps. The sequence of significant differences extended from 39.07 to 100% of movement time. For horizontal axis spatial terms, the bias toward the distractor was present as well (Fig. 15b), with 83 successive time steps showing significant differences at *p* < 0.01, exceeding the bootstrap criterion (*p* < 0.01) of six time steps. The sequence of significant differences extended from 45.70 to 100% of movement time. Similarly, for vertical axis spatial terms, the bias toward the distractor was present (Fig. 15c), with 71 successive time steps showing significant differences at *p* < 0.01, exceeding the bootstrap criterion (*p* < 0.01) of five time steps. The sequence of significant differences extended from 53.64 to 100% of movement time.

The right side of Fig. 15 visualizes the results of the comparisons by reference side, where red and blue circles labeled ‘R’ in the top of each panel indicate reference side for the correspondingly colored mean trajectory. Across spatial terms (Fig. 15d), trajectories diverged in a way consistent with a bias toward the reference. This effect spanned 36 successive time steps with significant differences at *p* < 0.01, exceeding the bootstrap criterion of four time steps. For this effect, the sequence of significant differences extended from 76.82 to 100% of movement time. The expected bias in spatial term direction in the first half of movement time was visible only as a non-significant tendency (the minimum *p* value within the first half was *p* = 0.023 at 30.46% movement time; *t*(23) = − 2.44). For horizontal axis spatial terms (Fig. 15e), the late divergence toward the reference item was more pronounced and became visible earlier. It was present over 77 successive time steps showing significant differences at *p* < 0.01, exceeding the bootstrap criterion (*p* < 0.01) of eight time steps. The sequence of significant differences extended from 49.67 to 100% of movement time. For vertical axis spatial terms (Fig. 15f), the early divergence consistent with a bias away from the reference (i.e., in spatial term direction) was significant. It was present over 20 successive time steps showing significant differences at *p* < 0.01, exceeding the bootstrap criterion (*p* < 0.01) of three time steps. The sequence of significant differences extended from 27.81 to 40.4% of movement time.

Condition-specific movement times are listed in Table 2. As in Experiment 1, *t* tests on movement time data showed no significant impact of distractor side, reference side, or spatial term axis (*ps* > 0.05). Similarly, there was no significant impact of spatial term axis on movement time difference scores between distractor sides or reference sides (*ps* > 0.05).

**Table 2:** Movement times and standard deviations (SD) for Experiment 2

	Distractor side				Reference side				Overall	
	Left		Right		Left		Right			
Spatial terms	Mean	SD	Mean	SD	Mean	SD	Mean	SD	Mean	SD
Overall	1061	127	1058	129	1058	123	1060	133	1059	126
Left/Right	1066	130	1065	137	1064	132	1066	137	1065	133
Above/Below	1058	121	1050	124	1055	114	1053	130	1054	122

### Discussion

The results were largely analogous to those of Experiment 1. Mean accuracy was marginally lower, but the target item was still chosen in the vast majority of trials. There were slightly more trajectories exceeding curvature threshold, potentially reducing the quality of estimated means due to less trials entering analyses. The distribution of curvature was not bimodal, however, and had a similar shape as in Experiment 1.

As hypothesized, a distractor effect was found in all comparisons by distractor side. The effect was overall comparable to its counterpart in Experiment 1, although its onset occurred somewhat later than before, and it was somewhat weaker in effect sizes and mean differences. This confirms the generality of the distractor effect. Furthermore, both a reference effect and a spatial term effect were found in comparisons by reference side. Most importantly, the hypothesized switch of the two effects with respect to the spatial term axes for which they occurred was observed. Attraction toward the side of the reference item was observed for horizontal axes spatial terms, as opposed to Experiment 1 where this effect was seen for vertical and not for horizontal axes spatial terms. Conversely, an early bias into the direction described by the spatial term occurred for vertical axes spatial terms, as opposed to Experiment 1 where this effect was seen for horizontal but not for vertical axes spatial terms. This switch confirms, first, that the attraction to the reference item is a general effect not dependent on spatial term axes or response direction, and, second, that a spatial term effect is exerted by both types of spatial terms, those with a horizontal axis, and those with a vertical axis. The switch also confirms that the spatial term was not a repulsion from the reference item.

Note that both the onset time and the magnitude of the reference effect were reduced compared to Experiment 1; in Experiment 2, it became significant only after approximately half of the total movement time. A more pronounced difference to Experiment 1 arose for the spatial term effect, which in Experiment 2 became significant only shortly after movement onset (while its end occurred at a time more similar to Experiment 1). This also affected the comparison by reference side across spatial terms, where the spatial term effect did not become significant. However, a trend toward significance in the earlier portion of the comparison by reference side for vertical axis spatial terms was present, suggesting that there was no fundamental difference between the spatial term effect observed here and in Experiment 1.

Finally, the between-subjects standard deviation of trajectories was overall considerably larger than in Experiment 1, as is obvious from comparing Figs. 11 and 15. Why this occurred is unclear; the low variability in Experiment 1 may have been a pattern based on chance, especially given the relatively low number of participants in Experiment 1 (and also because subsequent experiments showed a degree of between-subjects variability that was more similar to Experiment 2). Note that what mattered for the statistical comparisons was not the between-subjects variability of the compared mean trajectories, but the between-subjects variability of difference scores, which did not differ markedly between the two experiments (as can be derived from the figures by relating the difference between mean trajectories to effect size).

In summary, although the effects were somewhat weaker in Experiment 2, they were generally in line with Experiment 1. The hypotheses were borne out, suggesting that the three effects are universal across spatial terms and response metrics. This lends further support to the notion that the reference effect and the distractor effect were based on attraction toward visual items involved in spatial language grounding, and that the spatial term effect represented an influence of the semantics of linguistic spatial terms on motor action.

## Experiment 3

While the preceding experiments showed the biasing impact of individual visual items, this experiment focused on the effect of an additional relational pair. The goal was to investigate whether a biasing influence exerted by a second relational pair would transcend the sum of attraction caused by an additional reference item presented alone and by a distractor item presented alone. This would suggest that when items form a relational pair, a different or extended set of processes occurs than when items are presented in isolation. In turn, this would generally support that attraction observed in the experiments here were signatures of flexible cognitive grounding processes that varied with the grounding scenario and involved operations beyond attentional selection. It would argue against effect origins based purely on the impact of individual items, such as attraction toward mentioned colors.

**Fig. 16:**
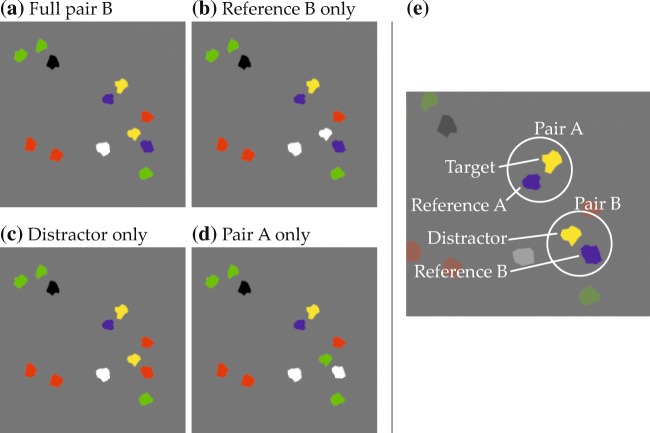
**a**–**d** Scene examples for each condition in Experiment 3 (only the stimulus region is shown). Panel (**e**) summarizes the involved items and terminology. All panels correspond to the spatial phrase “The yellow item to the right of the blue item”

As before, visual displays included a relational pair, labeled *pair A* in the following, which instantiated the spatial term from the relational phrase and was composed of a reference item (*reference A*) and a target item. In addition, each display contained a second relational pair, called *pair B*, which was identical to pair A except for being flipped along the spatial term axis (Fig. 16a). Pair B thus instantiated the inverse of the spatial term at hand. Thus, the item in pair B with the same color as the target item posed a *distractor*. The reference item in pair B will be referred to as *reference B*. The terminology is summarized in Fig. 16e.

Two additional conditions were introduced that used the same visual displays as in trials with a full second pair, except that in one condition (*distractor only*; Fig. 16c) reference B was replaced by a filler item, and in the other condition (*reference B only*; Fig. 16b) the distractor was replaced by a filler. The condition with both reference B and the distractor present was labeled *full pair B* (Fig. 16a). A baseline condition (*pair A only*; Fig. 16d) consisted of the same trials but with both items of pair B replaced by fillers.

Expectations in this experiment were inspired by the dynamic field model of spatial language grounding, which demonstrates how grounding processes of varying complexity arise from distinct grounding scenarios that differ in the combination of spatial phrases and visual scenes. Specifically, we expected that the presence of items forming a potential referent pair for the spatial phrase would lead to more complex processes than items presented in isolation, with each non-filler item in the display potentially being brought into the attentional foreground multiple times and a subset of items being probed for the sought relation in each grounding pass. We thus hypothesized that the amount of attraction evoked by a relational pair composed of a distractor and reference B would be larger than the sum of effects over conditions where either only a distractor or only another reference item was added to the visual display. In other words, an effect based specifically on the presence of pair B was expected to manifest as an interaction between the two factors of reference B presence and distractor presence. For the case of the competing hypothesis being true, that is, if a purely item-based mechanism were responsible for the observed attraction, we expected that the individual attraction caused by each of multiple items would combine additively. A distractor item would contribute a specific amount of attraction, as would an additional item in reference color, and if both were present, the sum of the two individual contributions would be observed. Therefore, no interaction was expected in this case.

In summary, Experiment 3 compared the combined but relation-independent impact of a distractor and a reference item to their relation-based impact through examining whether the presence of one item moderated the impact of the other. If so, it could be concluded that attraction effects were based on flexible processes of spatial language grounding rather than stereotypical processes evoked by individual items. An accessory hypothesis tested in this experiment was that attraction toward an item in reference color would occur even if that item was not particularly close to an item sharing the target color. This would further support that an item that is not a potential movement target can influence motor planning solely based on its role in the grounding process. This was examined by assessing the impact of reference B within the condition *reference B only*.

### Methods

#### Participants

The 20 participants (ten female, ten male) were 23.3 years (SD = 3.4 years) old on average and received €15 for participation. One had learned German only at the age of 12.

#### Procedure

Responses were oriented vertically as in Experiment 1. Trials with incorrect (i.e., non-target) responses were repeated once at the end of the trial list, to increase the amount of usable data and under the assumption that most incorrect responses were based on random errors due to fatigue and similar momentary effects.

#### Visual scenes

**Fig. 17:**
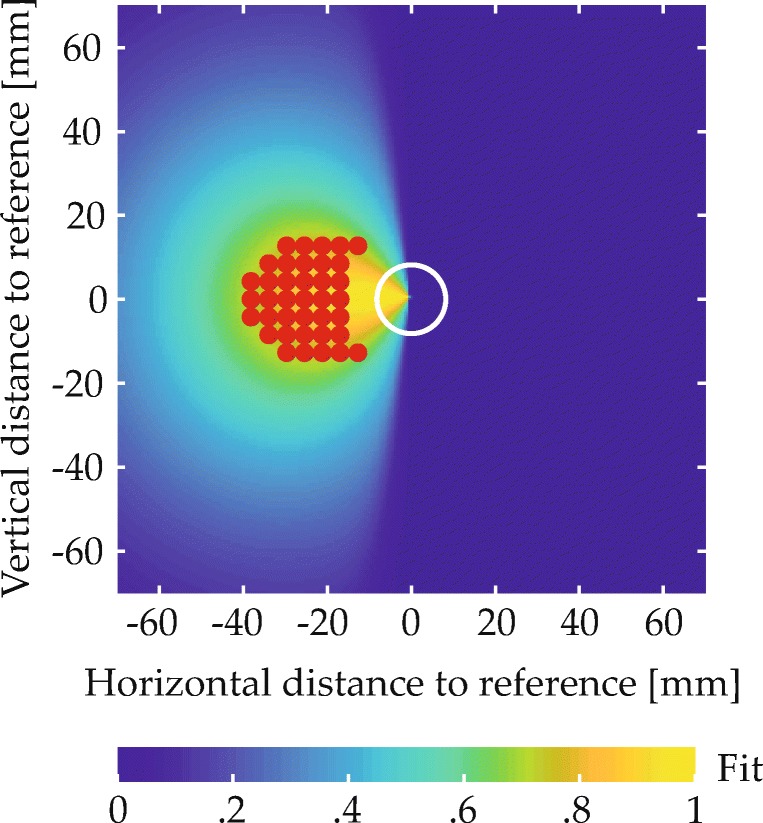
Possible target positions in Experiment 3. *Dots* mark target item centers, the *circle* marks the maximum reference radius

Scene creation followed similar principles as before; only new aspects are described. Target positions were located in a region with fit above 0.7 (rather than 0.6), to reduce the maximum spatial extent of pair A and leave more space for pair B. This resulted in 38 pair A configurations for each of the four spatial terms (illustrated for “left” in Fig. 17).

For each of these configurations, pair B was obtained by mirroring pair A along the spatial term axis (item shapes for pair B were determined randomly). Pair B was always placed entirely (i.e., both items) on the right or on the left side of the direct path, so that it could be treated as a single item during analysis. For each pair B side, 1280 trials were created, distributed equally among the four spatial terms (320 for each combination of spatial term and pair B side), and likewise equally distributed among the four possible target locations (320 for each combination of target location and pair B side). In half of the trials of each set of 320, reference A was to the left of the direct path and to the right in the other half (this facilitated later counterbalancing of the effect of reference A to isolate the effect of pair B). Within each set of 320 trials, the 32 pair A configurations were reused multiple times.

**Fig. 18:**
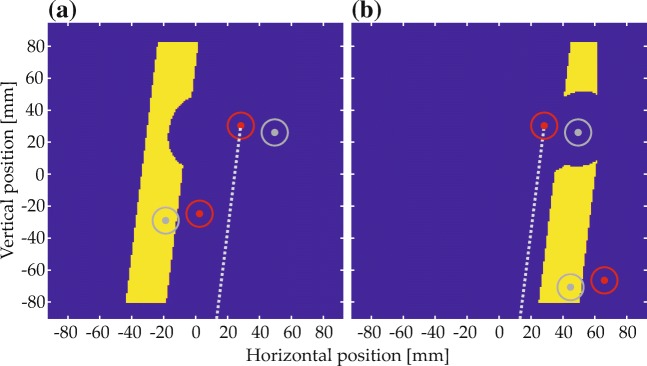
Example for possible pair B positions in the stimulus region, for spatial term “left”. Given the depicted relational pair, reference B could be placed in the yellow areas without violating placement constraints pertaining to the distractor or reference B itself. *Upper circles* represent the target (*red*) and reference A (*gray*). *Lower circles* represent the distractor (*red*) and reference B (*gray*), whose position in the figure represents one possible placement. **a** and **b** show templates for placing pair B left or right of the direct path, respectively. The *dotted gray line* is the direct path (from start marker to target center)

The exact placement of pair B on the desired side was random apart from the following constraints. Item centers were situated between 11.2 and 56.8 mm (0.92 to 4.64^∘^ v.a) from the direct path, to ensure that any attraction would produce measurable deviation while at the same time preventing participants from ignoring pair B due to its remoteness from the usual target locations. Two constraints prevented interference between pair A and pair B. First, when applying the spatial term relative to reference A, distractor fit had to be at least 0.25 lower than target fit. Second, target fit had to be at least 0.25 higher when applying the spatial term relative to reference A than when applying it relative to reference B. Combining all of the above constraints defined a region of eligible pair B locations for each combination of target location and pair A configuration. Figure 18 shows one example.

In total, this amounted to 2560 visual scenes. Fillers were added to arrive at 12 items in each scene (no opposing item in target color was used in this experiment). Each participant was assigned 128 scenes in a random manner but ensuring that the overall ratio of trial numbers in the different sub-conditions was preserved on the participant level.

Lastly, the scenes were modified to realize the four new experimental conditions. For each participant, the assigned set of 128 trials was reused in each of the four conditions in modified form, so that each participant had to complete 512 trials in total. The condition *full pair B* was represented by the unmodified displays. For the condition *distractor only*, the color of reference B was randomly changed to a filler color, so that only the distractor item remained of pair B. For *reference B only*, the color of the distractor was changed to a filler color, leaving only reference B. For *pair A only*, the colors of both the distractor and reference B were changed randomly and independently to filler colors, leaving only pair A.

#### Analysis

The main focus of the current experiment was to probe for an interaction effect of distractor presence and reference B presence on the dependent measure of trajectory divergence. Trajectory divergence was defined as the difference in trajectory deviation between trials where the present pair B items were located to the right of the direct path (pair B right) and those where they were located to the left of it (pair B left). It was computed by subtracting mean deviation in pair B left from that in pair B right, so that positive divergence indicates a bias in the direction of pair B and negative divergence indicates a bias away from it. This was done at each time step and within each of the four experimental conditions. The conditions were based on the factors *reference B presence* and *distractor presence*, each with the levels *present* and *absent*, and were named *full pair B*, *reference B only*, *distractor only*, and *pair A only*. Table 3 summarizes factors and conditions of this 2 × 2 within-subjects design.

**Table 3:** Conditions in the 2 × 2 within-subjects design of Experiment 3

	Distractor	
Reference B	Present	Absent
Present	Full pair B	Reference B only
Absent	Distractor only	Pair A only

The time-series of divergence values were compared between conditions by subjecting cell means at each time step to two-way repeated-measures ANOVAs. The employed alpha level was *p* < 0.04, which retained an overall alpha level of *p* < 0.05, since apart from the ANOVAs, one additional planned comparison was conducted with *p* < 0.01. This latter comparison used trajectory deviation as the dependent variable (as in the preceding experiments) and assessed the effect of reference B side within the condition *reference B only*, by comparing reference B right to reference B left.

Mean movement times within each condition (across pair B sides) were subjected to an ANOVA analogous to those used for the trajectory data (i.e., two-way repeated measures with factors *reference B presence* and *distractor presence*, but using *p* < 0.05) in order to explore whether any effects seen in the trajectory data would be reflected in movement times as well.

### Results

**Fig. 19:**
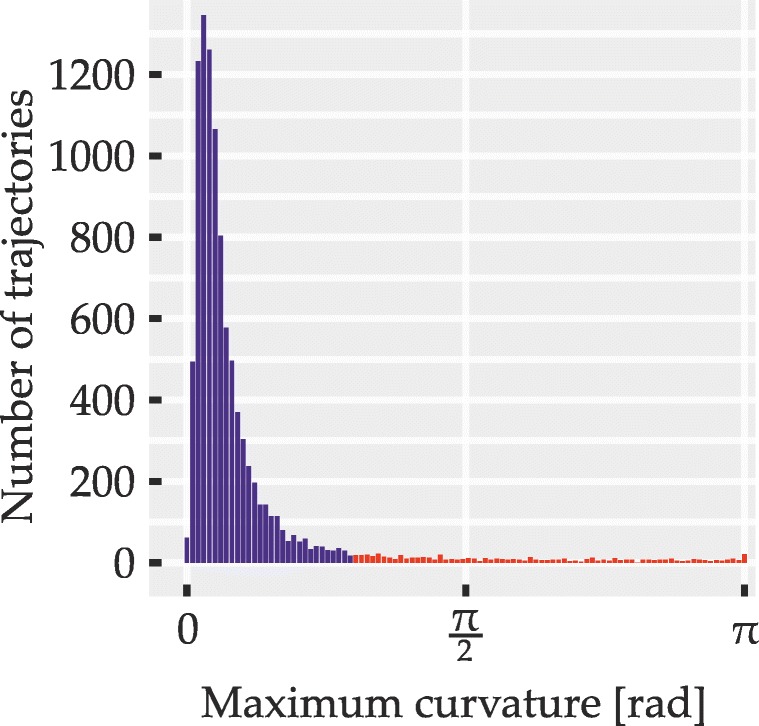
Distribution of trajectories over maximum curvature values in Experiment 3. *Red bars* correspond to trajectories that were discarded due to high curvature. Only correct responses are shown

A total of 10,240 trajectories was obtained. Of these, 9554 (93.3%) were below curvature threshold (M = 477.7, SD = 18.36 equaling M = 93.3*%*, SD = 3.59*%*).^4^ Of the non-curved trajectories, 98.17% (9379) were correct responses and thus entered further analysis (91.59% of all obtained trajectories). Movement onset was generally close to the center of the start marker (M = 1.72 mm, SD = 1.84 mm). Participants achieved a mean accuracy of 98.17% (SD = 1.89*%*) and their mean movement time was 1101 ms (SD = 121 ms). The above numbers are based on simple averaging over the respective trial ensembles; mean data reported from here on is based on balanced means as described. Figure 19 shows the empirical distribution over maximum curvature values for all correct responses, with red bars indicating curvature above threshold (i.e., trajectories excluded from other analyses). For the distribution Hartigan’s dip test indicated no bimodality (*p* > 0.05).

**Fig. 20:**
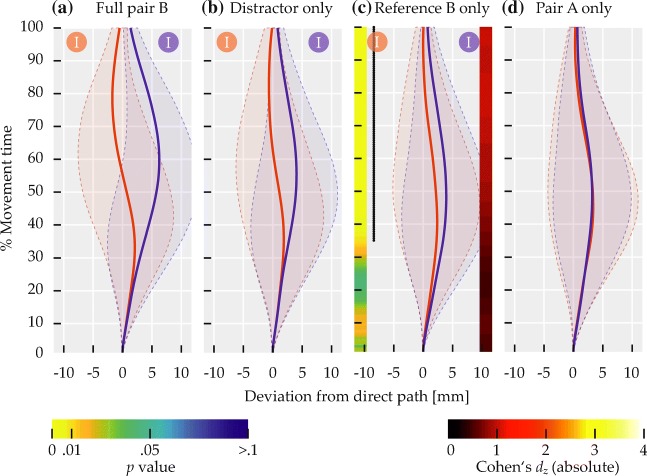
Deviation from the direct path in Experiment 3 for each condition and for each side of the item of interest (pair B as a whole, the distractor, or reference B). *Solid red and blue lines* show mean trajectory data, with *red and blue circles* labeled ‘I’ in the top of the panels indicating the side of the item of interest for the correspondingly colored trajectory. The image map on the left side of (c) shows *p* values, the right one effect size, and the *dotted black line* spans significant time steps (*p* < 0.01). Transparent regions delimited by *dashed lines* indicate between-subjects standard deviation

To provide a sense of the deviation toward items of interest in this paradigm, Fig. 20 shows mean deviation for each condition and for each side of the item of interest (pair B as a whole, the distractor, or reference B).

The comparison of left and right mean trajectories within condition *reference B only* (Fig. 20c) was significant, and indicated an extensive bias toward reference B with an early onset. It spanned 99 successive time steps showing significant differences at *p* < 0.01, exceeding the bootstrap criterion (*p* < 0.01) of four time steps. The sequence of significant differences extended from 35.1 to 100% of movement time.

**Fig. 21:**
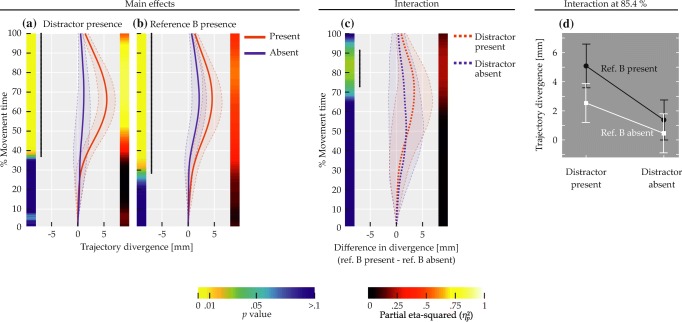
Result of the ANOVAs performed on trajectory divergence scores from experiment six. **a** Main effect of distractor presence. **b** Main effect of reference B presence. For (**a**) and (**b**), *solid lines* indicate mean divergence when the respective item was present (*red line*) versus absent (*blue line*), where positive divergence equals attraction toward the respective item. **c** Interaction of the two factors, plotted as the mean impact of reference B presence on trajectory divergence when the distractor was present (*red dashed line*) versus absent (*blue dashed line*). **d** Standard interaction plot for the point in movement time at which the lowest *p* was observed. Transparent regions delimited by *dashed lines*, and *error bars* in (**d**) indicate standard deviation between participant means. Note that *x*-axis scaling in this figure differs from previous ones that showed deviation

Figure 21 shows the results of the repeated measures ANOVAs of trajectory divergence with the factors distractor presence and reference B presence. The main effect of distractor presence (Fig. 21a) was significant at 96 time steps, the sequence extending from 37.09 to 100% of movement time, but the sequence length did not reach the criterion obtained from the bootstrap (based on overall *p* < 0.01) of 100 steps; however, this was likely due to the bootstrap method being prone to yielding overly conservative criteria in the case of particularly large effect sizes such as the one observed for this effect.

The main effect of reference B presence (Fig. 21b) was significant at 109 time steps, the sequence extending from 28.48 to 100% of movement time. This effect did exceed the bootstrap criterion for overall significance (based on overall *p* < 0.01) of 101 time steps.

The interaction between reference B presence and distractor presence (Fig. 21c) was significant as well, spanning 29 time steps, the sequence extending from 72.85 to 91.39% of movement time. The interaction exceeded the bootstrap criterion for overall significance (based on overall *p* < 0.01) of 13 time steps. Figure 21d shows an interaction plot for the time step at which the lowest *p* value was observed (85.43% movement time; *F*(1,19) = 5.669, *p* = 0.0279, ${\eta _{p}^{2}}=0.230$), to illustrate more clearly the stronger impact of “adding” one item to the display when the other one was present as well, compared to “adding” the same item to a display that did not contain the other item.

Condition-specific movement times are listed in Table 4, showing an apparent tendency of movement duration to increase in the order: *pair A only* (lowest), *reference B only*, *distractor only*, and *full pair B* (highest). This was corroborated by the ANOVA of movement time data, which showed significant main effects of distractor presence (*F*(1,19) = 63.909, *p* < 0.001, ${\eta _{p}^{2}}=0.771$) and reference B presence (*F*(1,19) = 37.77, *p* < 0.001, ${\eta _{p}^{2}}=0.665$) in terms of a movement time increase when the respective items were present. The interaction was not significant (*F*(1,19) = 2.643, *p* = 0.121).

**Table 4:** Movement times and standard deviations (SD) for each condition in Experiment 3

	Item of interest side					
	Left		Right		Overall	
Condition	Mean	SD	Mean	SD	Mean	SD
Full pair B	1130	124	1161	125	1145	121
Distractor only	1112	128	1114	125	1113	125
Reference B only	1076	125	1096	125	1086	124
Pair A only	1061	123	1073	115	1067	118

### Discussion

The main finding of Experiment 3 was the expected over-additive interaction between the presence of the distractor item and the presence of the additional reference item. Attraction was increased more strongly by placing a distractor in the vicinity of an item in reference color than by adding a distractor to a scene that otherwise contained only the sought relational pair. The analogue was true for adding an item that shared the reference color. This suggests that during spatial language grounding, additional processes take place when item combinations other than the pair described in the phrase may instantiate the sought relation by virtue of the items’ features.

Experiment 3 also showed that an additional item in reference color may attract trajectories even when there is no item in target color in its immediate vicinity. Interestingly, the effect was close in effect size and mean difference to the reference effects seen in previous experiments even though the additional reference item was placed more freely than the veridical reference item and thus tended to be more remote from the target and from the direct path. This may hint that participants did not move directly toward reference B but were gradually attracted toward its location by the postulated local enhancement of neural activation at its location. The effect also lends further support to the general interpretation of the reference effect as being independent of the spatial terms, since in contrast to reference items in the preceding experiments the placement of reference B was not coupled to the spatial terms.

Finally, movement times were increased both by the presence of the distractor and by that of the additional reference item. This mirrors the results from the trajectory data and likely stems from the fact that more pronounced attraction leads to longer mouse paths. Interestingly, the interaction between the two factors was not significant for movement times, suggesting that trajectory measures may be more sensitive to certain cognitive factors than movement time data, which is in line with previous hints in that direction (Koop & Johnson, 2011).

In summary, Experiment 3 suggests that, compared to isolated items in target or reference color, item pairs which pose potential referents for a spatial phrase lead to additional grounding processes that operate on a neural map of the task space.

## Experiment 4

The results of Experiment 3 were interpreted as reflecting cognitive grounding processes evoked by a pair of items posing a potential referent for the spatial phrase. An alternative interpretation is that any two potentially task-relevant items on the same side of the direct path may interact to cause a degree of attraction that transcends the sum of the items’ individual effects. This could not be ruled out completely based on Experiment 3 since it did not include a condition where two additional items were present on the same side of the direct path without forming a relational pair. This was probed in Experiment 4.

**Fig. 22:**
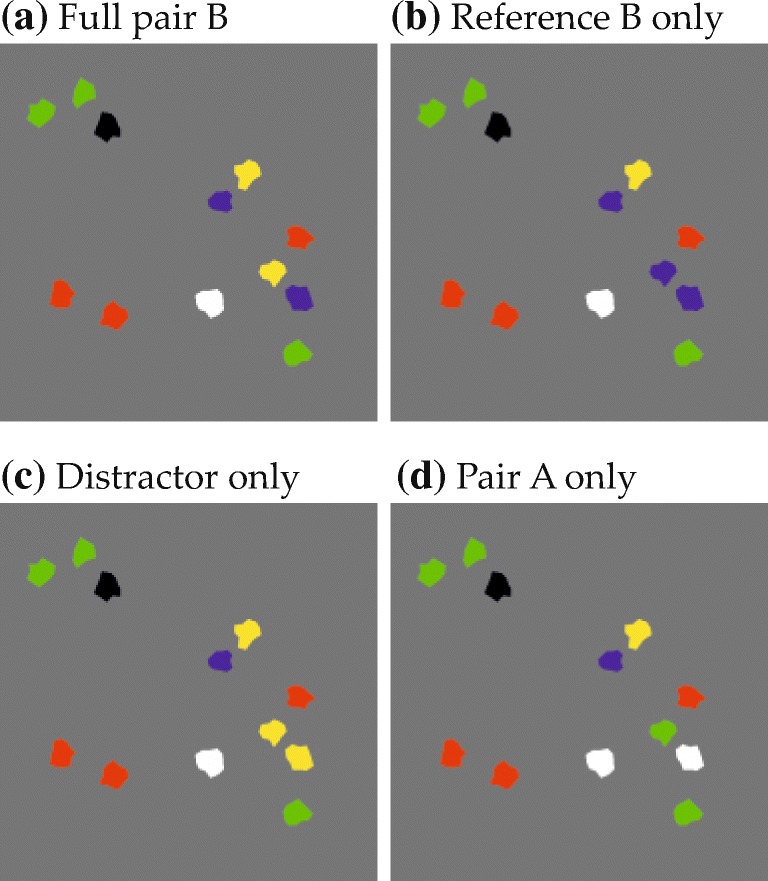
Scene examples for each condition in Experiment 4 (only the stimulus region is shown). The spatial phrase for these scenes was “The yellow item to the right of the blue item”

The only difference to Experiment 3 was that in the conditions *reference B only* and *distractor only*, the removed pair B item was replaced not by a filler color, but by the color of the remaining pair B item. That is, in the condition *reference B only* both items shared the color of reference A (Fig. 22b), and in the condition *distractor only* both items shared the color of the target item (Fig. 22c). Hypotheses were based on the assumption that the interaction in Experiment 3 was indeed the result of additional grounding processes. It was thus hypothesized that trajectory divergence between pair B sides would be stronger in the condition *full pair B* than in the condition *distractor only* (two distractor items) and stronger than in the condition *reference B only* (two reference B items).

### Methods

#### Participants

The 20 participants (11 female, nine male) were 27.1 years (SD = 7.7 years) old on average and received €15 for participation.

#### Visual displays

Visual scenes were the same as in Experiment 3 except that ‘absent’ pair B items were now assigned the same color as the remaining pair B item rather than being turned into a filler.

#### Analysis

As in Experiment 3, the dependent measure of trajectory divergence was computed for each time step and within each condition. The resulting time-series of difference scores were compared for the pairings *full pair B* versus *distractor only* and *full pair B* versus *reference B only*, each comparison using *p* < 0.01.^5^ For the same two pairings, movement times were compared by paired-samples *t* tests, each using *p* < 0.05 due to their exploratory nature.

### Results

**Fig. 23:**
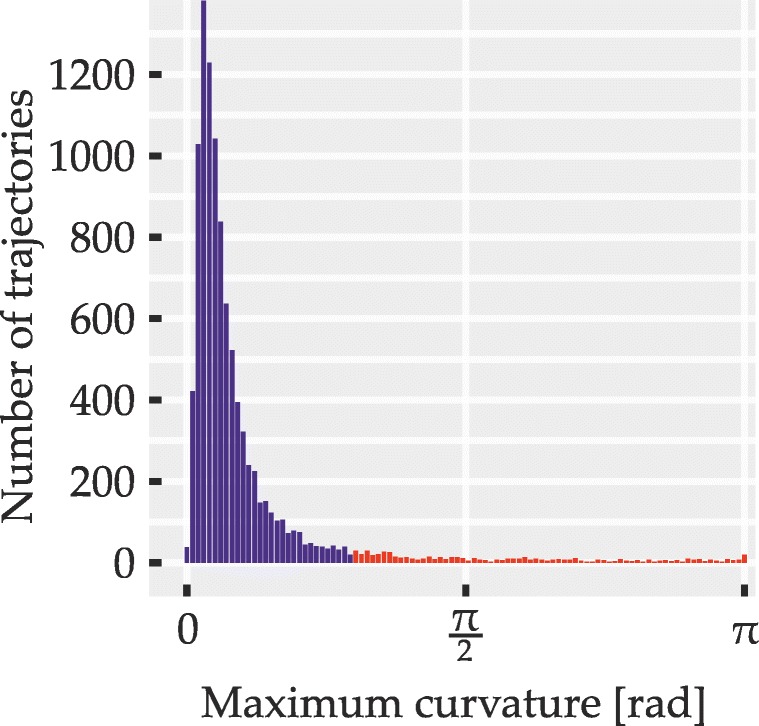
Distribution of trajectories over maximum curvature values in Experiment 4. *Red bars* correspond to trajectories that were discarded due to high curvature. Only correct responses are shown

A total of 10,240 trajectories was obtained. Of these, 9529 (93.06%) were below curvature threshold (M = 476.45, SD = 18.63 equaling M = 93.06*%*, SD = 3.64*%*).^6^ Of the non-curved trajectories, 98.79% (9414) were correct responses and thus entered further analysis (91.93% of all obtained trajectories). Movement onset was generally registered close to the center of the start marker (M = 1.84 mm, SD = 3.12 mm). Participants achieved a mean accuracy of 98.77% (SD = 2.61*%*) and their mean movement time across conditions was 1074 ms (SD = 125 ms). The above numbers are based on simple averaging over the respective trial ensembles; mean data reported from here on are based on balanced means as described. Figure 23 shows the empirical distribution over maximum curvature values for all correct responses, with red bars indicating curvature above threshold (i.e., trajectories excluded from other analyses). For the distribution, Hartigan’s dip test indicated no bimodality (*p* > 0.05).

**Fig. 24:**
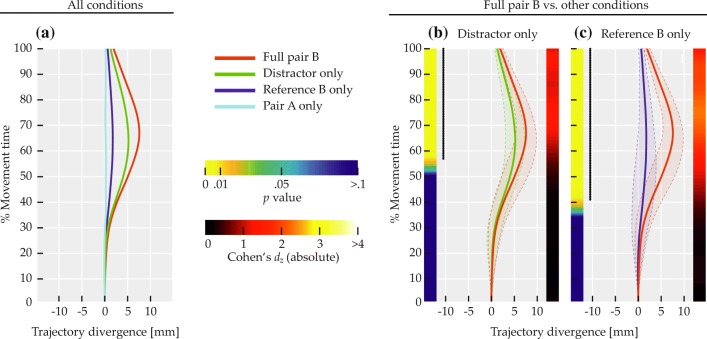
Mean trajectory divergence in Experiment 4. **a** All conditions in comparison. **b***Distractor only* versus *full pair B*. **c***Reference B only* versus *full pair B*. *Solid lines* show mean differences in trajectory deviation between the conditions ‘item of interest right’ and ‘item of interest left’. Positive values are consistent with attraction toward the item of interest, which was either pair B as a whole (*red lines*), two distractors (*green lines*), or two additional reference items (*dark blue lines*). The *light blue line* corresponds to the condition where only pair A was present. *Transparent regions delimited by dashed lines* indicate between-subjects standard deviation. *Left image maps* indicate *p* values, those on the right indicate effect size. *Black dotted lines on the left* span time steps with significant differences (*p* < .01)

Figure 24a shows mean trajectory divergence (item of interest side right minus left) for each of the four conditions.

Figure 24b shows the comparison of the condition *distractor only* (green line) to *full pair B* (red line), revealing that trajectory divergence was larger in *full pair B*. This difference was significant over 66 time steps, exceeding the bootstrap criterion of 32 time steps and extending from 56.95 to 100% of movement time. Figure 24c shows the comparison of divergence in *full pair B* (red line) and *reference B only* (blue line). In this case, the difference became significant earlier and was larger, with 90 time steps showing significant differences, thus exceeding the bootstrap criterion of 88 steps, and extending from 41.06 to 100% movement time.

Condition-specific movement times are listed in Table 5. Movement times were significantly larger in *full pair B* than in *reference B only* (*t*(19) = 9.66, *p* < 0.001, *d*_*z*_ = 2.16, mean difference 69.7 ± 32.3 ms) but not than in *distractor only* (*t*(19) = 1.94, *p* = 0.067, *d*_*z*_ = 0.43, mean difference 14.4 ± 33.1 ms).

**Table 5:** Movement times and standard deviations (SD) for each condition in Experiment 4

	Item of interest side					
	Left		Right		Overall	
Condition	Mean	SD	Mean	SD	Mean	SD
Full pair B	1111	138	1123	125	1117	130
Distractor only	1098	133	1107	132	1103	131
Reference B only	1046	112	1049	135	1047	122
Pair A only	1043	121	1041	118	1042	119

### Discussion

Most importantly, the trajectory divergence caused by an additional relational pair was larger than both trajectory divergence caused by two distractor items and trajectory divergence caused by two items sharing the reference color. This suggests that the increased attraction toward the relational pair seen here and in Experiment 3 was not based on a generic interaction between multiple task-relevant items situated in close vicinity to each other. It thus strengthens the notion that the interaction observed in Experiment 3 was indeed due to more complex processes of spatial language grounding as a result of the presence of an additional relational pair.

Movement time analyses mirrored the pronounced trajectory difference between an additional relational pair and two items sharing the reference color, which is again plausible as stronger deviation is associated with more distance traveled. There was, however, no difference in movement times between the additional relational pair and two distractor items, suggesting once more that trajectory measures may be superior to movement times in detecting subtle behavioral effects.

It has to be noted that while the double-item manipulation is more comparable to the presence of a full relational pair than are single items, it is still different with respect to the number of additional task-relevant colors. Since the only task-relevant colors are those of the target and the reference, no condition exists in which two additional task-relevant items are present that *differ* in color without forming a relational pair. In consequence, mechanisms associated with color representation cannot be ruled out completely as the origin of the differential impact of a relational pair and double items, although explaining the interaction seen in Experiment 3 on this basis would require further assumptions.

In summary, Experiment 4 supports that the stronger attraction observed toward a relational pair was based on more complex grounding processes.

## General discussion

In this study, participants moved a mouse cursor to a visual target item that was placed among other items in a visual scene. The target item was specified by a spatial phrase which described its color and its relation to a reference item. In addition, scenes could contain distractor items, which shared the target color but matched the relation to a lesser degree. Some scenes also contained more than one item in reference color. Differently colored fillers surrounded these items. To identify the target, the phrase must be grounded in the scene, which involved finding items in the colors mentioned by the phrase and assessing their spatial relation. Motor planning of the response movement was forced into the same time window as the grounding process by time-locking scene onset to movement onset.

We observed three effects that depended on where certain visual items were located in relation to the direct path (the straight line from start to target). First, the distractor effect was a trajectory bias toward the side of the direct path on which the distractor item was located. Second, the reference effect consisted of trajectory attraction toward the side of the direct path where the reference item of the spatial phrase was located; items that only shared the color of the reference exerted the same attraction. Third, increased attraction toward a competing relational pair was observed when two items in target and reference color were placed on one side of the direct path, located next to each other such that they instantiated the inverse of the sought relation. The presence of one item within this pair (e.g., the distractor) increased the amount of additional attraction caused by adding the other item to the display as well (e.g., the additional item in reference color).

The distractor effect can be interpreted along the lines of previous studies in which pointing trajectories were biased toward candidate targets when the target cue was delayed (Ghez et al., 1997; Gallivan & Chapman, 2014; Chapman et al., 2010). In the current study, the distractor shared the target color, so that participants likely viewed it as a potential movement goal until the phrase was fully grounded. For the reference effect, this interpretation does not apply. Color-based visual search likely allowed ruling out items in reference color as potential movement goals early after display onset. We therefore conclude that the attraction toward these items was not simply due to ongoing competition between motor decisions for movements toward potential targets, but was based on the involvement of the items in the cognitive process of spatial language grounding. The reference effect is thus of a different nature than previously reported influences on motor planning, which were either of a bottom-up perceptual or abstract cognitive origin. Based on this interpretation, we conjecture that similar mechanisms may also have contributed to the distractor effect.

Further support for the above considerations comes from the heightened attraction toward a competing relational pair, as it strongly suggests that effects were based on flexible cognitive grounding processes whose complexity increases when more potential referents for a given spatial phrase are present, and which include steps beyond attentional selection. This is also consistent with psychophysical data that showed low efficiency of relation processing when multiple candidate pairs were present and suggests that guiding attention to the target pair is not all that is required to evaluate its arrangement (Logan, 1994). That these properties seem to apply to the attraction effects observed here in turn supports that they were indeed the product of organized and flexible cognitive processes operating within sensorimotor substrates—rather than resulting from phenomena that are rooted more uniquely in perception, such as color priming (Schmidt, 2002) or effects of target distribution (Chapman et al., 2010). That the increased attraction toward a competing pair was indeed due to grounding processes rather than a generic interaction between closely spaced items is corroborated by the fact that item pairs in a single task-relevant color produced attraction that was still weaker than the attraction evoked by a competing relational pair.

In addition to the item-based attraction, we found a spatial term effect. This was a bias in the direction described by the spatial term, starting so early after scene onset that it could not have arisen from visual items in the scene. Its nature thus differs from that of the item-based effects. Rather, it is reminiscent of classical embodiment effects of language understanding that are not tied to particular targets in space but are based on task-irrelevant motor activation evoked by the semantic content of language (Tower-Richardi et al., 2012; Zwaan et al., 2012; Glover & Dixon, 2002; Gentilucci et al., 2000).

### Relation to the model of spatial language grounding

In the dynamic field model of spatial language grounding (Richter et al., 2014a, b, 2017), cognitive processes operate on a continuous neural representation of the task space by inducing activation shifts within that representation. We explain the item-based effects reported above as resulting from an influence of these cognitive activation shifts onto motor decisions that simultaneously occur in the same task space. The specific expectation that this should be reflected as attraction toward all targets, distractors, and reference items was derived from how grounding proceeds in the model: A feature-attention mechanism increases local activation for all items that share the reference color, to select one of them for further processing, and an analogous process occurs for items in target color. That this is mandatory arises from the neural restrictions (Schneegans et al., 2015a, b, Schneegans 2016) and attentional constraints (e.g., Franconeri et al., 2012; Hyun et al., 2009; Logan, 1994; Treisman & Gelade, 1980), which guided model creation. The expectation that the enhancement of local activation would lead to motor attraction was based on previous experimental and model-based evidence for sensorimotor coupling (Cisek, 2007; Cisek & Kalaska, 2005; Bastian et al., 1998; Erlhagen & Schöner, 2002). The observed attraction effects have confirmed this prediction.

The particularly strong effect of a competing relational pair as well is in line with the dynamic field model of spatial language grounding. In the model, scenarios with multiple possible candidate pairs may make several grounding attempts necessary, which include relational processes beyond attentional item selection, such as spatial transformation and repeated spatial term matching. Multiple sequential grounding passes and the associated relational processes increase the total activation that item positions receive over time, which may be at the basis of a stronger influence on motor decision-making compared to isolated items. It remains to be determined whether a distractor item and an item in reference color need to be spatially close to each other to be treated as a candidate for the sought pair and thus give rise to increased attraction. In the current state of the model, spatial distance between items in target color and items in reference color does not affect whether they are relationally assessed. Should future behavioral experiments reveal that spatial distance does matter, additional model mechanisms may be required to capture this, such as localized attentional biases, which ensure that only neighboring items are tested for the sought relation.

The activation in the perceptual field reflects the attentional requirements of the ongoing cognitive task because it is influenced by biasing inputs from feature search and relational components. It has to be noted, however, that it does not directly connect to a motor system. Such an extension of the model would be a valuable next step, as it would allow to directly compare model behavior to the human movement trajectories. In previous work, a neural representation similar to the perceptual field has indeed been used to drive a robotic arm toward visual targets (Knips et al., 2014; Tekülve et al., 2016; Zibner et al., 2015). However, the way in which movement was generated in these studies was not entirely biologically realistic, which is in fact required to afford a meaningful comparison with human movement. Realizing such an integrated model involves tackling multiple non-trivial issues, such as transformations between retinal and end-effector coordinates, generating the timing of motor movements, a realistic muscle model, and coordination of the arm’s multiple degrees of freedom. While possible in principle, such an endeavor is more than a trivial model extension (but see Lepora & Pezzulo, 2015, for a model that aims to link perceptual decision-making to motor action in a less process-oriented manner). Driving eye movements based on activation shifts in the perceptual field is another possible extension, which may generate further insights about the possible role of saccade patterns during spatial relation processing (Yuan et al., 2016; Burigo & Knoeferle, 2015). Existing modeling efforts (Trappenberg et al., 2001; Wilimzig et al., 2006; Kopecz & Schöner, 1995) suggest that a synaptic link of the perceptual field to a model of the neural structures for saccade generation like the superior colliculus is feasible.

Finally, as described above, the spatial term effect is probably of a different nature than the attraction based on localized visual items. As it does not appear tied to visual items, it lies outside the current scope of the dynamic field model. The spatial term semantics are in fact instantiated as an activation pattern within the model’s relational component (see Richter, Lins, Schneegans, Sandamirskaya, & Schöner 2014a, b; Richter et al. 2017 for details), providing a potential source for biasing movement accordingly. However, this pattern currently only serves to select the best-fitting target item from among eligible candidates within the relational component, and activation is enhanced in the perceptual field only at the selected item location. Adjusting the model to account for the spatial term effect on this basis would thus require re-thinking the connectivity structure between the relational component and the perceptual field. Whether this makes biological sense, and which exact adjustments would be required, is difficult to pinpoint as long as it is unknown from which neural systems the spatial term effect arises (e.g., it may arise from the motor side of the involved neural substrates rather than being perceptually based).

### Relation to similar effects

The effects we have observed are related to previously reported experimental evidence of various nature. First, there is evidence that stimuli which capture attention attract movement trajectories (e.g., Moher et al., 2015; Welsh, 2011; Wood et al., 2011). This is consistent with the interpretation of the distractor and reference effects as signatures of attentional allocation, which is captured in the dynamic field model as increased local activation.

Second, there is the rich body of mouse tracking research in which abstract cognitive tasks had to be solved and candidate solutions were linked to response locations in an arbitrary manner (e.g., Barca & Pezzulo, 2012; Dale et al., 2007; Freeman & Ambady, 2011; Freeman et al., 2013). Explaining the distractor effect along similar lines would mean that which item satisfied the relational phrase best might have been computed in substrates different from those where sensorimotor decisions are made. Ongoing competition between candidate solutions—target and distractors—would then have been linked and continuously streamed to item locations within a motor representation of the task space. In this view, the observed attraction would have arisen from the evolution of task processing over time elsewhere than in the immediate sensorimotor representations.

A first argument against this interpretation is that we varied the locations of movement targets from trial to trial^7^ and revealed them to the participants only upon movement onset. Neural decisions associated with motor planning and spatial language grounding thus had to occur in the same narrow time window. The coupling of abstract task solutions to response locations would thus have occurred rapidly and in parallel to the specification of the response movement. Effects began early after movement onset, especially in relation to the time required for color-based visual search, leaving little time for such coupling to occur. These constraints did not apply to previous studies, which typically showed response options in advance and at fixed positions. Others did use variable response locations but showed them before movement onset (e.g., Scherbaum et al. 2013; 2016) or coupled the presentation of targets or task information to movement onset without varying response locations (e.g., Dshemuchadse et al., 2013; Scherbaum & Kieslich, 2017). A second argument is that the reference effect differs in nature from deviation toward candidate task solutions, because the reference item was not a valid response option. It is thus difficult to attribute the attraction toward it to the same origin as the attraction toward response alternatives in previous mouse tracking studies.

Third, reach trajectories can be attracted by color primes that share a prespecified target color and are presented briefly prior to the veridical target, but at positions incongruent with the final target location; for instance, a red prime flashed in an upper position will gradually attract a trajectory that ultimately goes to a red target in a lower position (Schmidt, 2002; Schmidt & Seydell, 2008). Attraction to distractor and reference items may be framed along similar lines when assuming that color words mentioned in a spatial phrase have a similar effect as the target-defining instructions in these studies. Reference and distractor could then have played a role similar to the prime stimuli. There are indeed studies where two simultaneously relevant target-defining colors both lead to attentional capture by accordingly colored non-target stimuli (Moore & Weissman, 2010; Irons et al., 2012). However, these studies did not measure movement trajectories and did not explore what the joint effect of two simultaneously present and differently colored non-target stimuli may be, as would be required to explain the current data. More importantly, in priming studies and similar experiments, the target-defining feature is by definition consistent with the correct response, making it highly relevant, whereas here only one of the colors named in the spatial phrase was the color of a valid target and even this target was not specified unambiguously by the phrase. It is therefore questionable whether priming or similar effects played a decisive role in the current experiments, especially with respect to the reference item that was never a valid response. Moreover, the finding of greater attraction based on a competing relational pair poses additional problems to be explained in this manner.

Finally, it has to be noted that the different experiments described above, including ours, likely drew on heavily overlapping mechanisms that are part of a unified neural system which is involved in various sensorimotor and cognitive tasks. Plausibly, the shared nature of the neural mechanisms that are involved in seemingly different tasks may make it difficult—and sometimes impossible in principle—to fully dissociate the origins of similar effects. The similarity of the effects observed here to previously reported behavioral signatures can therefore be viewed as supporting rather than refuting the notion that spatial language grounding, and maybe other higher cognitive tasks, operate on sensorimotor representations and thereby influence the evolution of motor decisions.

### Conclusions

We have described a new mouse tracking paradigm that allows measuring effects of multiple variably positioned sources within complex visual scenes. This enabled us to assess the motor impact of different types of items in a complex cognitive task of spatial language grounding. In part, observed effects were similar to those seen in previous studies where potential targets attracted movement trajectories. The most surprising effects, however, could not be explained along these lines, but point toward mandatory processing steps of the ongoing grounding task as their cause. We have thus provided an important step toward unraveling the link between higher cognitive function and evolving activation in sensorimotor substrates.

#### Open practices statement

We make data associated with this paper available on our website at www.ini.rub.de. None of the experiments were preregistered.

## Figures and Tables

**Fig. 25:**
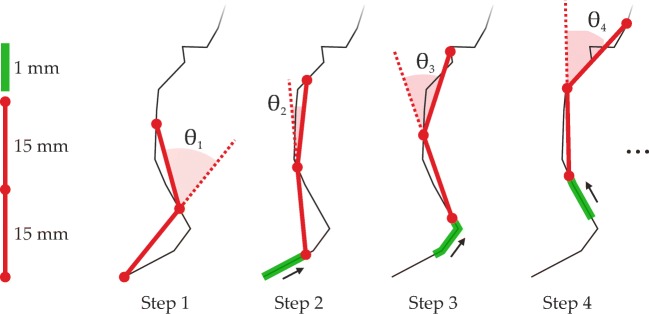
Four steps of the algorithm used for computing trajectory curvature, along with the parameters used for step size (*green bar*) and segment length of the fitted fragment (*red solid lines*). The *black line* represents a hypothetical trajectory. The *red dotted line* and the *region shaded red* represent *𝜃*_*i*_, the angle obtained in each step. Illustrations are not drawn to scale

**Fig. 26:**
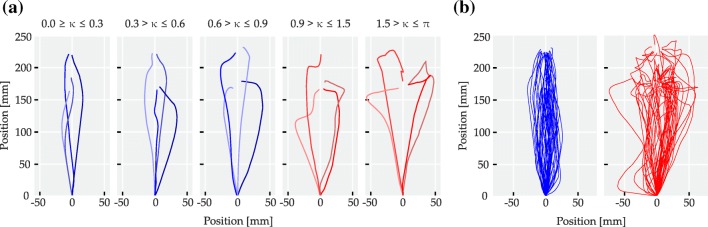
**a** Four example trajectories for each of five ranges of maximum curvature, *κ*. *Lines in shades of blue* represent trajectories included in the main analyses, *lines in shades of red* show trajectories that exceed the curvature threshold and were thus excluded from the main analyses. **b** Each plot shows 50 randomly selected trajectories obtained in Experiment 1 that are below curvature threshold (*left*) and exceed curvature threshold (*right*). Note that trajectories are plotted over spatial coordinates (not over percent movement time) since curvature computation as well operated on spatial coordinates and to better display what kind of trajectory shapes lead to exclusion

## Appendix A: Relational fit functions

The fit function *f*(*ϕ*,*r*), was defined over angle *ϕ* and radius *r*, providing a fit value for each spatial position around a reference item located at the coordinate origin. In polar coordinates, the function was given by

1 $$  f(\phi,r) = e^{\left[{-\frac{(\phi-\phi_{0})^{2}}{2\sigma_{\phi}^{2}}}\right]} \cdot e^{\left[{-\frac{(r-r_{0})^{2}}{2{\sigma_{r}^{2}}}}\right]} \cdot \left( 1\!+e^{\beta(|\phi-\phi_{0}|-\phi_{\text{flex}})}\right)^{-1}, $$

where *ϕ* denotes polar angle, *r* is the radius, *ϕ*_0_ is the mean of a Gaussian function over angle, *σ*_*ϕ*_ is that Gaussian function’s standard deviation, *r*_0_ and *σ*_*r*_ are analogous parameters for a Gaussian over radius, *β* is the steepness of a sigmoid function over angle, and *ϕ*_flex_ is the separation of its inflection point from the mean of the Gaussian over angle. The parameters used were *σ*_*ϕ*_ = 1.05, *r*_0_ = 0 mm, *σ*_*r*_ = 47 mm, *β* = 25, and *ϕ*_flex_ = 1.45. The parameter *ϕ*_0_ differed between spatial terms, with “right of”, “above”, “left of”, and “below” corresponding to $\phi _{0}=\{0,\frac {\pi }{2},\pi ,\frac {3}{2}\pi \}$ radians.

The function shapes were inspired by behavioral data (Logan & Sadler, 1996; Hayward & Tarr, 1995) that has been reproduced by a computational model based on similar functions (Lipinski et al., 2012).

## Appendix B: Determining trajectory curvature

The algorithm used to determine trajectory curvature was applied to the non-interpolated spatial trajectory data. In summary, it worked by fitting a three-point line fragment (shown in red on the left side of Fig. 25) with two segments of fixed length (15 mm each) to a given trajectory at successive positions. The resulting angles between the two segments were used as a measure for local curvature. Figure 25 shows four steps of the procedure for hypothetical trajectory data (black line). The procedure started by placing the fragment’s first point on the first data point (Fig. 25, step 1). Next, the fragment’s central point was placed at a subsequent location on the trajectory while keeping the first point stationary (in case of multiple possible placements, the one at the shortest arc length along the trajectory was chosen). Lastly, while again keeping the first two points stationary, the third point was placed on a location further down the trajectory. Since segment length was fixed, this required adjusting the angle between the first and the second segment of the line fragment. The resulting angle between the first and the second segment was used as first local curvature value (region shaded red and labeled *𝜃*_1_ in Fig. 25, step 1). In the next step (Fig. 25, step 2), the fragment’s first point was shifted along the trajectory by a step size of 1 mm (green bar in Fig. 25), followed by the same fitting procedure as before to place the other two points, thus obtaining a second local curvature value, *𝜃*_2_. This was repeated using the same step size of 1 mm until the end of the trajectory was reached. The maximum curvature, *κ*, for a given trajectory was determined by $\kappa =\max \limits (\{\theta _{1},\dots ,\theta _{n}\})$, where *n* denotes the total number of angles obtained for the trajectory.

The advantage of this procedure is that the obtained measure of local curvature is independent of the varying Euclidean length of trajectory segments in the raw data. The outcome is thus largely independent of the temporal resolution at which mouse movement is sampled. Furthermore, tuning the parameters of the algorithm (step size and segment length) allows adjusting the scale of trajectory turns that contribute to curvature, so as to suit the nature of the data at hand. For instance, a larger segment length decreases the impact of small deviations from the principal trajectory direction and at the same time assigns higher curvature to larger turns formed by many short trajectory segments that change direction only gradually. The parameter values used for the present data set (segment length of 15 mm and step size of 1 mm), as well as the exclusion threshold (0.933 radians), were initially tuned as described in the analysis section and then used throughout the reported analyses. Figure 26 provides an impression of the outcome of the curvature computation and exclusion procedure using the final parameter values, with Fig. 26a showing trajectory exemplars for successive ranges of maximum curvature (*κ*; trajectories exceeding the threshold are shown in red) and Fig. 26b showing a larger number of examples for included (blue) and excluded (red) trajectories from Experiment 1 (patterns in the other experiments were very similar).

## Footnotes

1 A preliminary report of Experiment 1 has been published in non-archival form in Lins and Schöner (2017).

2 Although numerically this does not fully account for the six comparisons, it was deemed sufficient given that contrasts were preplanned and hypothesis-guided (Armstrong, 2014), and due to the non-independence of data sets underlying some of the contrasts, which tends to increase the conservativeness resulting from correction procedures (Winer et al., 1991; Abdi, 2007)

3 A preliminary report of Experiment 2 has been published in non-archival form in Lins and Schöner (2018).

4 Note that, over participants, an average of 42.5 (SD = 19.33) trials were presented twice due to incorrect responses in the first presentation, as described earlier. If the second presentation of a trial was responded to correctly, only this response was included in the remaining analyses.

5 *p* < 0.01 was used since three accessory comparisons were conducted, which we omit for brevity here.

6 Note that an average of 39.05 (SD = 21.48) trials per participant were presented twice due to incorrect responses in the first presentation, as described earlier. If the second presentation of a trial was responded to correctly, only this response was included in the remaining analyses.

7 The location of the correct target was in fact variable between only four positions across trials, but this was successfully masked by the position of all other items being highly variable (as described earlier, participants reported not to have noticed that targets could occur at only four locations).
